# Wearable E‐Textiles for Cardiovascular Monitoring: Materials, Devices, and Integrated Systems

**DOI:** 10.1002/advs.77999

**Published:** 2026-11-04

**Authors:** Bangul Khan, Mahmoud Tavakoli, Norbert Radacsi, Bee Luan KHOO

**Affiliations:** ^1^ Department of Biomedical Engineering College of Biomedicine City University of Hong Kong Kowloon Tong Hong Kong; ^2^ Hong Kong Centre of Cerebro‐Cardiovascular Health Engineering (COCHE) Shatin Hong Kong; ^3^ Institute of Systems and Robotics Department of Electrical and Computer Engineering University of Coimbra Coimbra Portugal; ^4^ School of Engineering Institute for Materials and Processes University of Edinburgh Edinburgh United Kingdom; ^5^ School of Engineering Institute for Bioengineering University of Edinburgh The King's Buildings Edinburgh United Kingdom; ^6^ Centre for Cardiovascular Science The Queen's Medical Research Institute (QMRI) University of Edinburgh Edinburgh United Kingdom; ^7^ Center of Excellence for Biosensors and Bioengineering (CEBB) Chulalongkorn University Bangkok Thailand; ^8^ Institute of Digital Medicine City University of Hong Kong 83 Tat Chee Avenue Kowloon Tong Hong Kong

**Keywords:** cardiovascular monitoring, ECG, E‐textiles, intelligent wearables, smart textiles

## Abstract

Cardiovascular diseases remain the leading cause of morbidity and mortality worldwide, highlighting the need for continuous, non‐invasive, and reliable physiological monitoring beyond conventional clinical assessments. Smart textiles have emerged as a promising platform for personalized cardiovascular healthcare by combining wearability, mechanical comfort, and seamless integration into everyday garments. This review examines recent advances in conductive materials, textile manufacturing technologies, and system‐level device integration for cardiovascular sensing applications. Metallic fibers, carbon nanomaterials, and conducting polymers are evaluated for electrical conductivity, stretchability, mechanical durability, and wash stability. Furthermore, textile fabrication approaches, including weaving, knitting, embroidery, printing, and electrospinning, are analyzed for their ability to translate material properties into scalable, manufacturable sensing architectures. Recent developments in textile‐integrated electrocardiography, pulse‐wave, blood pressure, and hemodynamic monitoring demonstrate performance approaching clinical standards, with high signal fidelity and strong agreement with conventional medical devices. Key challenges related to long‐term stability, power autonomy, signal reliability, and large‐scale manufacturing are discussed alongside emerging solutions, including MXene‐based electrodes, self‐powered sensing systems, and edge artificial intelligence. Finally, future perspectives are outlined for developing clinically validated, scalable, and intelligent smart textile platforms that enable continuous cardiovascular monitoring and next‐generation personalized healthcare.

## Graphical abstract

The graphical abstract highlights the role of conductive materials and textile fabrication techniques in enabling smart textiles for cardiovascular applications. It showcases textile‐based ECG monitoring, pulse sensing, hemodynamic monitoring, and cardiovascular tissue engineering, while emphasizing the challenges and design considerations that govern device performance, wearability, and clinical translation toward personalized healthcare.

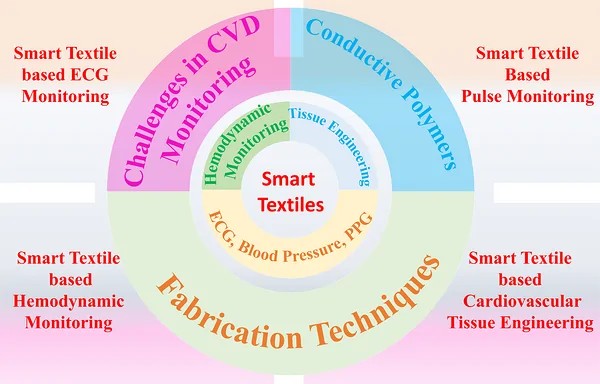

## 1 Introduction

Cardiovascular diseases (CVDs), including hypertension, myocardial infarction, coronary artery disease, and heart failure, remain the leading cause of morbidity and mortality worldwide. The increasing prevalence of these disorders highlights the urgent need for effective strategies for early detection, continuous monitoring, and timely intervention. Despite advances in cardiovascular care, delayed diagnosis and inadequate long‐term monitoring continue to create significant clinical and economic challenges on healthcare systems globally [1, 2].

The development of cardiovascular monitoring systems is moving significantly beyond traditional Holter monitors, bulky electrocardiogram (ECG) devices, blood pressure monitors, and photoplethysmography devices toward Artificial Intelligence‐based wearable systems. Traditional CVD devices were effective for precise, accurate detection, but their bulky nature limits their use to clinical settings. Additionally, the time constraints, economic cost, and comfort were also major concerns [3, 4]. AI‐enabled wearable technologies elevate healthcare systems by enabling early detection and diagnosis of cardiovascular diseases. Wearable cardiovascular devices provide long‐term wireless monitoring of critical heart parameters, including ECG, Blood pressure, and SpO_2_, with comfort, helping healthcare professionals monitor heart status without a significant time and economic toll. In addition to their advantages, wearable devices face challenges related to flexibility, accuracy, and mechanical‐electrical trade‐offs [5, 6, 7].

In the 20th century (the 1990s), Steve Mann, an MIT researcher, attempted to integrate textiles with rigid electronics, a concept later investigated by several researchers over the decades, ultimately leading to the development of wearable smart textiles, also known as e‐textiles [8, 9]. Smart wearable textiles have been explored in various forms, including fibers, conductive fabrics, coated fabrics, and yarns that incorporate sensing and conductive properties. These smart textiles have been well explored in biomedical engineering, extending beyond sensing and actuation. Compared to traditional wearable devices, such as smartwatches and fitness bands, e‐textiles offer greater adaptability, flexibility, and comfort for all users. They are easier to customize in terms of size and color and provide larger sensing surfaces, enabling multiple sensor placements across the body rather than being limited to specific spots, such as the wrist or chest [10, 11].

Despite significant advances in conventional wearable technologies, including smartwatches, chest straps, and adhesive medical patches, several limitations still limit their suitability for long‐term cardiovascular monitoring [12]. Smartwatches are typically confined to the wrist and primarily rely on optical sensing, which may suffer from motion artifacts and limited signal fidelity during daily activities. Chest straps can provide more accurate physiological measurements but are often uncomfortable for prolonged wear and may discourage long‐term user compliance [13, 14]. Similarly, adhesive medical patches offer high‐quality physiological recordings but can cause skin irritation, decreased adhesion during perspiration, and discomfort during extended monitoring periods [15, 16]. In contrast, e‐textiles can be seamlessly integrated into everyday garments, providing large‐area, skin‐conformal sensing without substantially altering user behavior. Their inherent flexibility, breathability, and ability to support distributed sensor networks across multiple body locations enable continuous, unobtrusive, and multimodal cardiovascular monitoring while improving long‐term wearability and user adherence. These characteristics position e‐textiles as a promising alternative for next‐generation personalized cardiovascular healthcare [17, 18].

The reviewed literature collectively underscores the transformative potential of smart textiles in healthcare, yet each article approaches the topic from a distinct perspective. W. Zhan et al. [19] emphasize material innovation and fabrication strategies, focusing on fiber electronics and their integration into textiles to achieve multifunctionality, breathability, and durability under mechanical stress. In contrast, N. Sunstrum et al. [20] explore disease‐specific applications, particularly in diabetes and cardiovascular care, advocating multimodal sensing platforms that integrate optical, biochemical, biomechanical, and thermal techniques to enhance accuracy and reliability in non‐invasive monitoring. Iftikhar Ali et al. [21] provide a comprehensive, design‐oriented review that addresses sensor selection, integration, signal processing, and user acceptance, while highlighting practical challenges such as calibration, biocompatibility, and scalability. Meanwhile, M. Azeem et al. [22] focus on energy autonomy, detailing advancements in energy‐harvesting technologies, particularly the role of 2D materials in thermoelectric, piezoelectric, and triboelectric systems, to overcome power limitations in wearable electronics. Although these studies provide valuable perspectives, most emphasize materials, fabrication approaches, or general wearable healthcare applications. Comprehensive reviews dedicated specifically to cardiovascular monitoring applications and their clinical translation remain limited.

This review provides a comprehensive overview of smart textiles for cardiovascular monitoring, beginning with the key cardiovascular physiological cues that serve as targets for continuous wearable sensing. Subsequently, the major challenges associated with cardiovascular monitoring, including electrode‐skin interfacing, signal reliability, wearability, and system integration, are discussed to establish the design requirements of smart textile platforms. The review then summarizes the conductive materials and textile fabrication techniques that enable the development of flexible and conformal sensing systems. Building on this foundation, recent advances in smart textile‐based electrocardiography (ECG), pulse‐wave monitoring, hemodynamic monitoring, and cardiovascular tissue engineering are critically examined. Finally, current performance trade‐offs, emerging technologies, and future directions are discussed to highlight the path toward clinically translatable and intelligent smart textile systems for continuous cardiovascular healthcare.

## 2 Cardiovascular Diseases and Physiological Cues

Cardiovascular Diseases (CVDs), such as hypertension, arrhythmias, and heart failure, remain the leading causes of death worldwide. The asymptomatic and intermittent nature of such disorders calls for continuous and long‐term physiological monitoring. Hypertension poses a great risk of causing stroke, heart attack, and kidney failure, while arrhythmias disturb the regular heart rhythm and can readily contribute to sudden heart failure. Heart failure, another type of CVD, further impairs heart function to effectively enable blood circulation, causing fluid retention, fatigue, and poor quality of life [23, 24].

Recent breakthroughs in Artificial Intelligence and sensor‐integrated textiles offer an encouraging alternative, as these wearable technologies enable non‐invasive, painless, and continuous measurement and monitoring of cardiovascular physiological signals, including heartbeats and blood pressure. In view of the increasing prevalence of CVD, which is expected to significantly affect the ageing population, the use of textile technology represents an important shift toward the practical and preventive management of cardiovascular disease [25, 26].

### 2.1 Crucial Cardiovascular Physiological Cues

Measuring cardiovascular function requires multiple physiological parameter. The assessment of cardiac electrical activity, arrhythmias, ischemia, and conduction problems still depends on the electrocardiogram (ECG). Electrocardiogram measurements are the centerpiece of textile‐based wearable systems because they directly capture the heart's electrical activity at each phase of the cardiac cycle. As can be seen from the (Figure 1Ia), the mechanical function of pumping by the heart, caused by contractions of the atrium and ventricles, is directly related to distinct ECG waveform (Figure 1Ib) components: the P wave reflects atrial depolarization and thus the electrical trigger for atrial contraction; the QRS complex has a sharp peak and is of high amplitude, reflecting rapid ventricular depolarization and thus the onset of forceful ejection of blood. The T‐wave activity following the QRS complex reflects ventricular repolarization, thus indicating electrical recovery before the next cycle. Parameters such as wave amplitude and duration, including the QT interval, provide important cues for detecting arrhythmias, ischemia, and conduction abnormalities [27]. Textile wearables that incorporate soft electrodes and conductive fabrics enable continuous, unobtrusive ECG monitoring in daily life, a powerful tool for early detection and long‐term management of cardiovascular conditions [11, 28].

**FIGURE 1:**
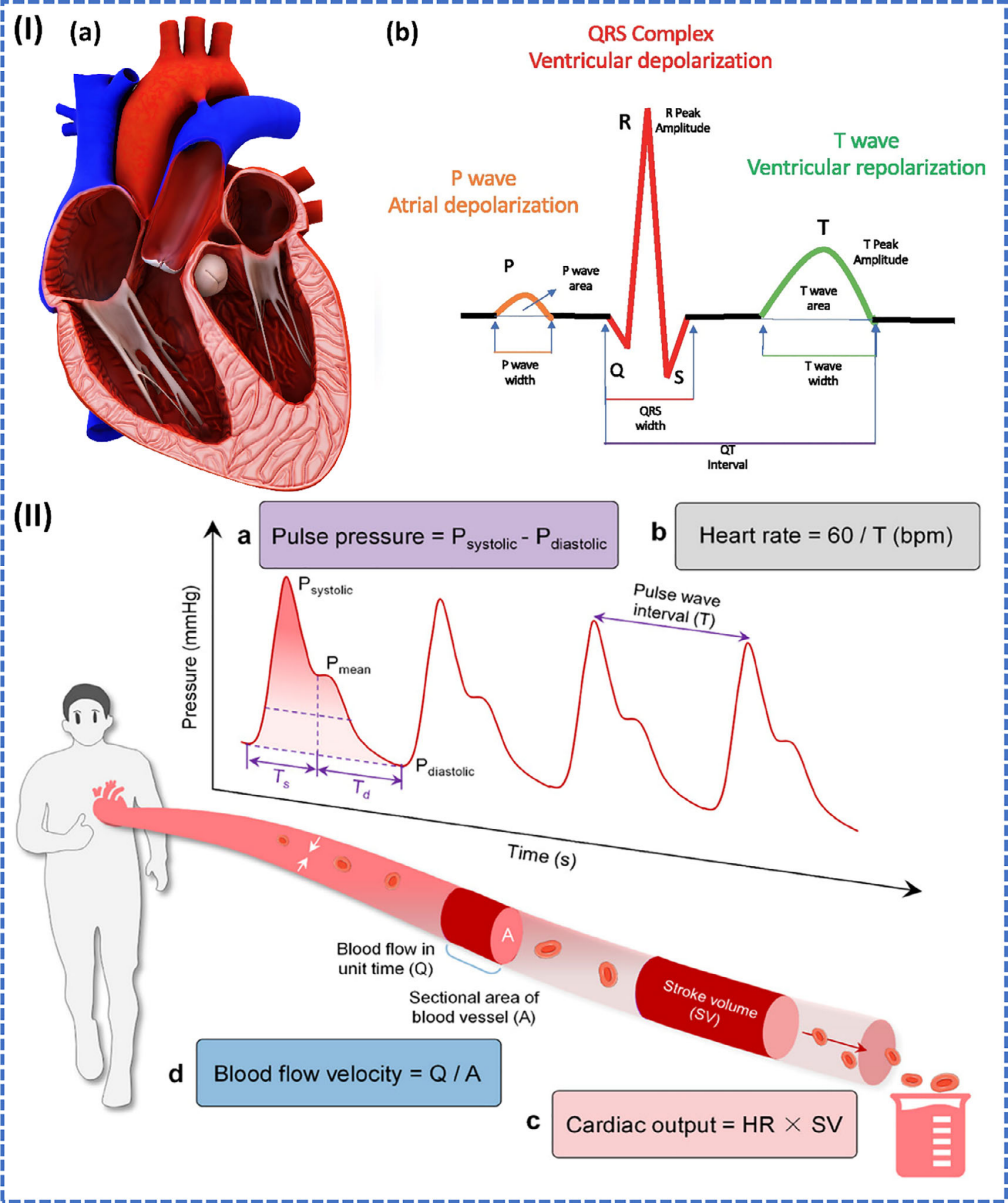
Crucial Cardiovascular Physiological Variables (I) Heart anatomy and key ECG waveform components. (a) Anatomy of Cardiac structures and blood‑flow pathways, (b) illustration of the ECG waveform, highlighting the P wave (atrial depolarization), QRS complex (ventricular depolarization), and T wave (ventricular repolarization). These electrical features provide essential cues for wearable textile‑based cardiac monitoring. Subfigure b reprinted from [27] under Creative Commons CC‐BY‐NC license. (II) Key physiological indicators for hemodynamic monitoring. (a) Blood pressure (mmHg) and the associated arterial pulse waveform, with systolic and diastolic pressures identified. (b) Heart rate is defined as the number of cardiac cycles per minute (bpm). (c) Cardiac output represents the volume of blood pumped by the heart each minute. (d) Blood‑flow velocity indicates the speed at which blood moves through the vascular system. Reprinted from [37], Creative Commons CC BY license.

Real‐time ECG measurement with textile electrodes yields high‐quality ECG signals and greater comfort than with gel electrodes. Heart Rate (HR), measured using ECG signals or Photoplethysmography (PPG) techniques, reflects autonomic function and exercise capacity [29, 30]. Blood Pressure (BP) is a crucial parameter used to indicate Hypertension. Textile sensors using piezoresistive or capacitive techniques could enable continuous BP measurement without the need for cuffs [31, 32]. Pulse Wave Velocity (PWV), a well‐known marker of arterial stiffness, is highly sensitive to cardiovascular diseases and correlates with morbidity and mortality. In textile‐based systems, PWV is typically determined using a combination of ECG, PPG, IPG, or pressure sensors positioned at different body locations. PWV is calculated as the difference in transit times between sensors, thereby providing important information about vessel status and arterial elasticity [33, 34]. All physiological inputs are needed to conduct a full analysis of the cardiovascular system and the integrity of blood vessels. Textile sensors, along with multi‐mode sensors, will enable integrated assessment of cardiac function, measurement of Heart Rate, BP, and PWV, and advanced computational analysis for diagnostic purposes [35, 36].

### 2.2 Hemodynamic Monitoring

Hemodynamics refers to the forces and fluid dynamics that underlie blood circulation and provide crucial insights into the physiology and pathology of the circulatory system. Unlike other liquids, blood circulates within a pulsatile, branched, compliant vascular bed and exhibits non‐Newtonian properties necessary for transporting oxygen and nutrients, as well as metabolic waste products. The biological and physical complexities of this process make continuous and precise hemodynamic analysis and monitoring both difficult and valuable. The heart's pumping action generates pulsatile pressure waves that drive systemic and pulmonary circulation. Arteries, veins, and capillaries maintain pressure gradients and facilitate gas and nutrient exchange.

The most important hemodynamic variables, including blood pressure, arterial and venous flow velocities, and vascular resistance, depend on vessel elasticity and geometry. Conventional methods of hemodynamic monitoring, such as sphygmomanometry or catheter techniques, enable intermittent or invasive hemodynamic analysis [37, 38, 39]. As depicted in Figure 1II, for efficient hemodynamic monitoring, four main parameters should be monitored: Blood Pressure (BP), Heart Rate (HR), Blood Flow Velocity (BFV), and Cardiac Output (CO). BP (Figure 1IIa) is measured in systolic, diastolic, and mean values, reflecting both arterial elasticity and cardiac function. Additionally, HR (Figure 1IIb), derived from the heart cycle, provides prompt feedback on autonomic function and workload. CO (Figure 1IIc) represents the volume of blood ejected per minute and indicates the heart's ability to deliver oxygenated blood; it can be estimated from arterial waveforms using mean arterial pressure and peripheral resistance. BFV (Figure 1IId) describes the speed of blood flow and depends on cardiac force, vessel diameter, resistance, and blood viscosity. Together, these four parameters offer a comprehensive view of cardiovascular function and serve as fundamental targets for textile‑integrated wearables designed for continuous hemodynamic monitoring [37].

### 2.3 Tissue Regeneration

Cardiovascular tissue engineering aims to restore, replace, or augment impaired cardiac and vascular tissues by integrating biomaterials, cells, and bioactive cues to reconstitute functional physiological milieus. Due to the poor regenerative capacity of the native myocardium and vasculature, human diseases like heart failure, myocardial infarction, and vascular disease increasingly depend on engineered constructs. Traditional strategies use hydrogels, polymeric scaffolds, and decellularised matrices as substitutes for the ECM to maintain cell adhesion and guide tissue organisation. However, obtaining mechanical properties relevant to physiology and electromechanical coupling, and ensuring long‐term stability, are challenging, especially in dynamic tissues such as the myocardium and arterial walls [40, 41, 42].

Recently, textile‐based scaffolds, such as woven, knitted, braided, and electrospun fibrous scaffolds, which draw inspiration from textiles, have emerged as a promising approach for the regeneration of the cardiovascular system. The anisotropic properties, high porosity, and structural integrity of textile materials make them well‐suited to withstand the cyclic deformations typical of the heart and blood vessels. Knitted, woven, braided, and warp‐knitted scaffolds offer the necessary properties for a myocardium and contractile cell alignment, as well as burst strength and compliance for a vascular graft [43, 44, 45].

The fibre mats created by electrospinning, which mimic the ECM perfectly, aid re‐endothelialisation, the integration of smooth muscle cells into vascular tissues, and accelerate smooth muscle cell maturation. In parallel, the application of hybrid textile‐integrated scaffolds made of conductive polymers, carbon nanotubes, or metallic microfibers enables the creation of an electrical pathway, which is crucial for the binding and synchronisation of cardiomyocytes [46, 47]. In addition to regenerative scaffolds, there are emerging applications in bioreactors and biologically based conditioning that provide mechanical cues, including stretching, flow simulations, and electromechanical signals, for tissue maturation. In these emerging applications, where there is greater integration and interaction between bioelectronics and engineered tissue, there appears to be a link between textile materials and sensing and stimulation components in more complex systems, such as biological systems involving cardiovascular tissue engineering [41, 48].

## 3 Challenges in Cardiovascular Monitoring

The continuous monitoring of the cardiovascular system plays a significant role in the early detection, diagnosis, and treatment of various deadly conditions like arrhythmia, hypertension, arterial stiffness, coronary artery disease, and heart failure. Although conventional systems are known to provide accurate results, the problem is their infrequent use, as tests are performed only in hospitals or specialized facilities. However, continuous monitoring using wearables has gained significant public interest, yet accuracy, wearability, and comfort remain challenges [49, 50].

The challenge of cardiovascular monitoring lies in the need to measure several physiological parameters simultaneously. An electrocardiogram (ECG) provides information about the heart's electrical activity and is of great importance in detecting arrhythmias and conduction abnormalities [51]. Measurement of the pulse wave is required to assess heart rate and vascular function; blood pressure (BP) and pulse wave velocity (PWV) are good markers of arterial stiffness [33]. Hemodynamic monitoring measures cardiac output, blood flow velocity, and vascular resistance, and is more accurate for assessing cardiovascular system performance [52]. To measure these parameters, multimodal sensing methods should be used, including ECG, PPG, IPG, pressure and strain measurements, and wireless data acquisition. Compliance with such multimodal clinical standards demands rigorous performance requirements for wearable devices [53, 54].

Accurate measurement of cardiovascular parameters depends on the stability of the skin‐to‐sensor interface, real‐time data acquisition, low impedance, high sensor responsiveness, and resilience to environmental conditions. Motion artifacts, sweating, changes in body position, and incorrect sensor placement may compromise physiological data collected during regular activities [55, 56].

The use of e‐textile technology offers considerable possibilities for addressing these problems, as it enables adaptation to human shape and the embedding of sensors in clothing. But the performance of fabric cardiovascular system devices depends not only on device architecture but also on the textile's design. The positioning of the electrodes and sensors, garment design, and pressure and sensing areas are important factors that affect the accuracy and reproducibility of the targeted data [57, 58]. Apart from detection methods, many other factors should be considered when designing such a system to achieve successful cardiovascular monitoring using e‐textiles. The continuous and stable functioning of cardiovascular monitoring devices requires effective power management, wireless transmission, on‐chip signal processing, and secure health information processing [8, 59]. The use of artificial intelligence, edge computing, and multimodal sensor fusion technologies has made the interpretation of physiological signals more effective and reduced noise. However, there are still technical problems related to computational capacity, power consumption, privacy, and clinical validation of the results [60, 61].

### 3.1 Electrode–Skin Interface Challenges

Electrode‐skin contact is the critical factor affecting the quality of the recorded signals in wearable cardiac monitoring devices. While there have been numerous attempts to create high‐conductivity materials, nanofibers, and textiles for use as electrodes, the signal quality is often determined by contact stability rather than by the electrode's conductivity. The electrode‐skin interface directly affects contact impedance, signal‐to‐noise ratio, waveform distortion, and motion artifacts in cardiovascular monitoring signals [62, 63].

Particularly in ECG monitoring, classical Ag/AgCl electrodes use conductive gel to reduce impedance and improve contact quality. In contrast, dry textile‐based electrodes rely primarily on direct physical contact with the skin. Hence, changes in garment fit, contact pressure, body movement, sweating, and skin topography can alter the electrode‐skin interface and degrade signal quality. High‐conductivity electrodes may still give very poor ECG results if there is no stable contact between the electrode and the skin [64, 65].

This problem becomes especially crucial when monitoring the pulse and heart over a long period, as the effects of stretching, body movement, and changes in body position can lead to baseline drift. All these factors can affect the accuracy of heart rate and arrhythmia detection.

Recently, more attention has been paid to interface engineering solutions to overcome these drawbacks rather than to the development of improved electrode materials. Methods such as textile design, embroidery and knitting of electrodes, use of compression clothing, surface structuring, use of flexible MXene fabric conformal electrodes, and pneumatic assistance in contact have been proposed to increase the device's conformability to the skin and preserve stable contact during movement [66, 67, 68, 69].

### 3.2 Textile Structure‐Function Challenges

The performance of cardiovascular monitoring systems based on textile sensors depends not only on the properties of the materials used in sensors and electronics but also on the structure of the textiles. Indeed, textile structure significantly influences the mechanical, electrical, sensor‐skin contact, and sensor performance. Still, the correlation between textile structure and the performance of wearable cardiovascular monitoring systems is a significant challenge [70, 71].

The mechanical behavior of textile structures varies; for example, woven fabrics show excellent dimensional stability and strength but are inextensible. For this reason, woven textiles are ideal candidates for applications requiring stable sensor fixation [72, 73]. Meanwhile, knitted fabrics exhibit high stretchability, flexibility, and conformability, enabling them to follow the body's contours and maintain stable skin contact. These properties are especially critical for continuous ECG and pulse wave recording [74, 75].

Additionally, the structure of the textile fabric can influence the electrical behavior of the textile electrode. Stretching, bending, and compression can affect the conductive paths in textile electrodes and their connections. As a result, there will be changes in resistance and signal instability. However, knitted structures can deform more readily; their electrical resistance changes under deformation, whereas woven structures have better electrical stability but reduced mechanical flexibility. Electrodes embroidered on textiles enable adjustment of the conductive pathway via density and stitch direction [76, 77, 78].

To conclude, the textile structure is the key element that connects the material's features to the device's performance. Future electronic textile devices can only become competitive if we consider not only the textile structure but also other elements, including materials and sensors.

The summarized cardiovascular techniques, physiological cues, possible diagnoses, and Challenges are highlighted in Table 1.

**TABLE 1:** Summary of the cardiovascular techniques.

Technique	Physiological Cues	Diagnoses	Challenges	References
ECG	Electrical activity (P, QRS, T)	Arrhythmias, ischemia, conduction issues	Motion artefacts, electrode contact issues	[79, 80]
Heart Rate (ECG/PPG)	HR, beat variation	Tachycardia/bradycardia, autonomic issues	Accuracy during motion, skin tone variability	[81, 82, 83]
Blood Pressure	Systolic/diastolic, pulse wave	Hypertension, arterial stiffness	Calibration drift, cuffless accuracy limits	[36, 83, 84]
Cardiac Output	Blood volume/min	Heart failure, pump deficiency	Indirect estimation errors	[85, 86]
Hemodynamic Monitoring	BP, HR, BFV, CO	Overall cardiovascular function	Multisensor integration complexity	[37, 87]
Tissue Regeneration	Mech/electrical cues	Myocardial repair, grafts	Biocompatibility, long‐term stability	[43, 88]

## 4 Materials for Smart Textiles

Smart textiles are combinations of materials, such as conductive components, biocompatible polymers, and hybrid composites, to trade off biocompatibility, stretchability, flexibility, and conformability [89]. This section discusses the conductive components, such as metallic fibers, carbon‐based structures, and conductive polymers, which are fundamental to signal transduction in electrocardiography, photoplethysmography, and pressure‐sensing devices.

### 4.1 Conductive Materials

Conductive materials are the cornerstone of smart textile technology, offering efficient signal transmission, making them ideal for cardiovascular sensing. Such conductive materials range from metallic fibers and carbon‐based materials to conductive polymers, each offering distinct benefits in electrical conductivity, availability, flexibility, and biocompatibility [90].

#### 4.1.1 Metallic Fibers

The metallic fibers contributed significantly to smart textiles due to their low‐resistance conductive pathways and stable electrode interfaces, which are necessary for acquiring high‐resolution physiological signals. Various metallic fibers have been reported in the literature, including stainless steel, silver‐coated nylon, copper, and gold‐coated filament [91]. Metallic fibers are selected based on factors such as conductivity, oxidation resistance, cost, and purity. Several fabrication techniques, including knitting, weaving, braiding, and embroidery, were employed to promote geometric freedom and ensure continuity with the skin even during patient mobility [92]. Pre‐treatments, such as silver plating, reduce contact impedance, which is ideal for high‐fidelity signal transmission [93]. The wearability of metallic electrode configurations is improved by a metal‐shell design that attaches metal components to a rigid polymer core, thereby suppressing metal degradation [94]. Additionally, elastomer protection that shields against corrosion makes it durable in a humid environment. Long‐term cardiovascular monitoring requires comfortable wear, which can be optimized by using different yarns and textile shapes that produce softer textures with reduced rigidity [95]. In the cardiovascular approach, motion interference poses a significant challenge in continuous, long‐term monitoring. Several researchers have developed serpent‐shaped conductive textile geometries on silicone substrates that form a conformal electrode capable of transmitting microvolt‐level signals even during patient ambulatory activity. Hence, metallic conductive pathways remain a popular choice when made wear‐friendly with adequate corrosion protection [96, 97].

#### 4.1.2 Carbon‐Based Materials

Carbon‐based materials, such as graphene, carbon nanotubes (CNTs), carbon black, and reduced graphene oxide (rGO), revolutionize smart textile technology by providing lightweight, flexible, breathable, and conductive characteristics [98]. The high aspect ratio of these carbon‐based materials, with a percolation mechanism that exhibits variable sheet resistance, makes the smart textile effective, precise, and accurate in monitoring cardiovascular signals. Additionally, spray coating, dip coating, inkjet printing, and in‐fiber growth of carbon‐based materials help develop a conformal layer that remains conductive even under flexure and stretching [99]. The electrochemical stability of graphene‐CNTs supports the design of impedance sensors for non‐invasive, wearable devices; modification (carboxyl‐ or amine‐functionalized) enhances interfacial charge‐transfer properties, an essential requirement for minimizing biofouling [100, 101]. The significant advantages of carbon‐based conductive nanomaterials are their low mass, fatigue resistance, and air‐permeable design, which help enable long‐wearing, comfortable devices [101]. The use of carbon‐based conductive nanomaterials in smart textile devices poses challenges. Still, the controlled use of flake size, purity, and the presence of a foil residue from a metal catalyst, wrapped with elastomers of a specified degree, helps develop a biocompatible environment [102, 103]. Additionally, carbon‐based materials are summarized in Table 2.

**TABLE 2:** Carbon‐Based Conductive Materials in Smart Textiles.

Material	Structure	Electrical Conductivity	Thermal Conductivity	Advantages	Disadvantages	References
Carbon Black	Amorphous carbon nanoparticles	0.1–10 S cm^−^^1^	Moderate	Low cost, stable	Lower conductivity	[104, 105, 106]
SWCNT	Single graphene cylinder	∼10^5^ S m^−1^	∼2000–3000 Wm^−^^1^K^−^^1^	Excellent conductivity	Expensive	[107, 108, 109]
MWCNT	Concentric nanotube layers	10^3^–10^5^ S m^−1^	∼3000 Wm^−^^1^K^−^^1^	Strong, durable	Dispersion difficulty	[107, 110]
Graphene	2D honeycomb sheet	>10^6^ S m^−1^	∼4000 to 5300 Wm^−^^1^K^−^^1^	Strong, flexible	Costly	[107, 111, 112]
Graphene Oxide	Oxidized graphene	10^−^^6^–10^−^^1^ S m^−1^	∼10–1000 Wm^−^^1^K^−^^1^	Easy dispersion	Lower conductivity	[112, 113, 114]
Reduced Graphene Oxide	Reduced GO	1.5×10^5^ S m^−1^	30–2600 Wm^−^^1^K^−^^1^	Flame‐retardant, conductive	Adhesion issues	[115, 116, 117]

#### 4.1.3 Conductive Polymers

Conductive polymers are 3D, highly flexible materials that offer greater flexibility, conductivity, and skin‐conformal properties, prerequisites for long‐term cardiovascular monitoring [118]. A wide array of conductive polymers is employed in wearable textiles, while Poly(3,4‐ethylenedioxythiophene) polystyrene sulfonate (PEDOT: PSS) is well known for its transparency, conductivity, and processability in aqueous solutions. It is ideal for layering on fabrics via ink deposition or dip‐coating methods [119, 120]. PEDOT: PSS's ionic‐electronic conduction mechanism is an advantage over other conductive polymers for making low‐resistance bio‐interfaces, enabling high‐quality physiological signals for early detection of cardiovascular diseases. PEDOT: PSS has been identified as one of the most effective conductive polymers for producing wearable textile‐based electrodes owing to its excellent conductivity, flexibility, and biocompatibility [121].

Polyaniline (PANI) has a unique chemical structure, is environmentally stable, and can be readily prepared in aqueous solution, endowing it with excellent film‐forming properties and making it one of the most widely used intrinsically conductive polymers for textiles [122]. PANI offers flexible, durable films that provide functionality from both mechanical stress and washing standpoints. Furthermore, it exhibits good electrical conductivity in its emeraldine salt state, strong adhesion to substrates, and tunable conductivity via acid doping and pH control [123]. PANI‐coated fabrics may serve as electrodes to monitor ECG and heart rate for continuous, non‐invasive signal acquisition during daily activities. Moreover, its electrochemical properties also enable sensors for assessing blood pressure and vascular health, which is crucial for early detection of cardiovascular conditions [124, 125].

Polypyrrole (PPy) is also a conductive polymer with a conductivity typically in the range of 1–100 S cm‐1, providing great flexibility for wearable and implantable textiles [126]. It is mostly deposited on textile fibres by chemical or electrochemical methods, yielding a robust film with controlled conductive properties and various doping agents (pTs), which are useful for strain‐sensitive components, dry electrodes, and other applications [127, 128]. Additionally, Table 3 summarises the properties of conductive polymers.

**TABLE 3:** Summary of the properties of conductive polymers.

Conductive Polymer	Key Properties	Fabrication Methods	Advantages	Challenges	Reference
PEDOT: PSS	Highly conductive (300–500 S cm^−1^), flexible, skin‐conformal	Dip‐coating, ink deposition, enzymatic polymerization, wet‐spinning	Low‐impedance bio‐interfaces, high ECG quality	Additive‐dependent conductivity, wash durability	[119, 129, 130]
Polyaniline (PANI)	Tunable conductivity (0.1–6 S cm^−1^), film‐forming, stable	In situ polymerization, coating, electrospinning, and blending	Flexible ECG electrodes, high sensitivity	Environmental instability, swelling, and doping are required	[131, 132]
Polypyrrole (PPy)	Highly conductive (0.1–100 S cm^−1^), flexible, biocompatible	Chemical/electrochemical deposition, coating, lamination	Self‐powered sensing, strain/pressure sensing	Brittle in pure form, adhesion issues	[126, 133, 134]
Polythiophene (PT)	Good optical & electronic properties, highly conductive (10^−2^ to 10^2^ S cm^−1^)	Chemical polymerization, vapor‐phase polymerization, coating	Tunable functionality for sensing	Low conductivity, low solubility, lower stability	[135, 136, 137]
Poly(p‑phenylene vinylene) (PPV)	Optical properties, stable, conductivity (10^−7^ S cm^−1^)	Chemical synthesis, precursor casting, and doping	High stability, good electronic behavior	Insoluble, requires doping	[138, 139, 140]

## 5 Fabrication Techniques

Fabrication methods are essential for incorporating functional materials into textiles, significantly impacting sensors, sustainability, and usability. Several fabrication techniques, including weaving, knitting, printing, coating, embroidery, and electrospinning, are explored for smart textile‐based wearable electronics for long‐term cardiovascular monitoring. This section discusses recent advancements in the mentioned fabrication techniques and their basic principles, with summarized characteristics presented in Table 4.

**TABLE 4:** Comparison of Fabrication Techniques for Smart Textiles.

Technique	Principle	Advantages	Limitations	Scalability	Cost	References
Weaving	Interlacing warp & weft yarns	Stable structure, reliable electrodes	Low stretch, rigid	High	Moderate	[159, 160, 161]
Knitting	Interlooped yarns	Stretchable, body‑conforming	Conductivity changes with strain	High	Low–Moderate	[161, 162, 163]
Embroidery	Stitching conductive threads	Precise placement, customizable	Wash durability varies	High	Low–Moderate	[162, 164, 165]
Printing	Depositing conductive inks	Lightweight, fine‑detail patterns	Ink cracking, wash limits	Very High	Low	[166, 167, 168]
Electrospinning	Nanofiber formation	Ultra‑thin, sensitive	Scaling challenges	Moderate	Moderate	[157, 169]

Weaving involves the orthogonal interlacing of warp and weft yarns to produce structurally stable fabrics with precise, customizable conductive pathways. Weaving with metallic or carbon yarns provides dimensional stability, making it particularly suited for use in ECG sensors and wearable electrodes [141]. Multi‐weaving is used to develop protective topologies, while Jacquard weaving is used to design complicated topological arrangements for sensors, with high malleability. This weaving technique helps provide consistent electrode positioning, thus eliminating motion artefacts [141, 142].

Knitting utilizes the continuous interlooping of yarns to yield highly elastic textile matrices. This inherent structural loop configuration ensures excellent mechanical stretchability and multi‐directional conformability. The knitted fabric is highly stretchable due to interloop yarn interlacing, providing excellent conformability. The use of conductive yarns in ribbing or interlock knits that closely conform to the skin minimises noise in cardiovascular monitoring [143, 144]. The use of spacer knits provides additional pressure‐sensitive areas, which are useful in sensing pulses, thus extending the applications of knitted structures to dynamic, ambulatory devices. The loop structure provides uniform strain distribution, thereby maintaining conductive paths during cyclic loading [144, 145].

Textile embroidery enables the precise, localized deposition of conductive threads onto pre‐existing fabric substrates, allowing for the direct integration of complex circuit layouts and localized medical sensing nodes. Embroidery can also be done using machine‐embroidered cloth, ranging from clothing to smart materials such as medical textiles [146, 147].

The development of e‐textiles for cardiovascular monitoring requires materials and fabrication approaches that simultaneously deliver high electrical conductivity, mechanical stretchability, and scalability. These requirements are difficult to satisfy with conventional conductive materials and have driven extensive research into stretchable conductive inks compatible with textile manufacturing processes, such as printing [148, 149].

Carbon‐based inks (e.g., carbon nanotubes, graphene, carbon black) are widely studied for their intrinsic flexibility, low cost, and printability. They can withstand large strains while maintaining relatively stable electrical performance, making them attractive for wearable systems. However, their electrical conductivity remains significantly lower than that of metallic counterparts, limiting their use in applications requiring high signal fidelity or low‐noise biosignal acquisition [150, 151].

Silver‐based inks offer substantially higher conductivity and are already widely used in printed electronics. Stretchable formulations based on silver flakes or nanoparticles embedded in elastomers provide initial compliance; however, under repeated mechanical deformation, these systems typically suffer from microcrack formation, percolation loss, and progressive increases in resistance, which can compromise long‐term reliability in wearable applications [152].

Recent advances in printed smart textiles demonstrate that screen printing and inkjet printing have emerged as potent fabrication routes for producing flexible, lightweight, and conformal electrodes and other electronic components suitable for cardiovascular monitoring [153, 154, 155].

Electrospinning is a nanofiber production technique in which a high voltage electric field is applied to stretch a polymer solution into thin fibers that solidify into a porous nonwoven fabric [156]. While it is a very simple process, it requires precise control over parameters such as the viscosity of the polymer solution, the applied voltage, the flow rate of the liquid polymer solution, solvent volatility, and humidity [157]. Large‐scale electrospinning without compromising fiber quality can be challenging. To address these challenges, various modifications of electrospinning techniques have been employed, including needleless, coaxial, and melt electrospinning, to increase productivity and functionality [158].

## 6 E‐Textiles for Physiological Monitoring

### 6.1 Smart Textile‐based ECG Monitoring

Electrocardiography is a crucial tool for early cardiovascular detection. Long‐term ECG monitoring is challenging due to mechanical, electrical, and biocompatibility trade‐offs associated with traditional electrodes [170]. Therefore, textile‐based electrodes emerge as a viable solution, offering higher flexibility, excellent biocompatibility, and precise signal acquisition. To advance textile‐based ECG electrodes, several teams are employing a range of techniques, designs, and synthesis methods to improve their performance [171].

Maithani et al. used the direct‐write laser‐induced graphene on a conductive Kevlar textile. The Kevlar textile was coated with PEDOT: PSS, followed by LIG. Maithani et al. developed electrodes that demonstrated excellent conductivity, with contact impedances ranging from 100 ± 3 kΩ to 7.9 ± 3 kΩ over 40Hz to 1kHz. Additionally, the developed electrodes produce high‐resolution ECGs for several hours without irritation, indicating their potential for clinical applications [172]. Terada et al. reported a structural fabric electrode that serves as an intrinsic noise‐reduction filter by creating a capacitive low‐pass filter through multiple fabric‐folding stages. It greatly enhanced the quality of ECG signals, achieving a signal‐to‐noise ratio (SNR) of about 12.02 dB compared to standard fabric electrodes [173]. Xue et al. demonstrated an electrochemical, porous, dry ECG electrode based on graphene nanoplatelets (GNPs)/polyamide 66 (PA66) that was incorporated into a personalized ECG shirt. The developed electrode had a low skin‐electrode impedance of 17.6 kΩ at 100 Hz and an ECG signal‐to‐noise ratio of 34.76 dB, which was 8% better than that of the commercially available Ag/AgCl electrodes [174]. To simplify the synthesis technique, Ferri et al. employed transfer printing of ECG electrodes, thereby enabling attachment to various types of garments and fibres. The performance of the electrodes was evaluated using the gold‐standard Ag/AgCl electrodes in 10 human volunteers, demonstrating SNR above 28 dB with high‐resolution ECG signals [175].

Reduced graphene oxide‐coated cotton textile (rGOC) electrodes were developed by Saleh et al., which outperformed Ag/AgCl electrodes in ECG applications. The optimized rGOC circular electrode exhibited an SNR of 14.85 ± 0.22 dB compared with that of the Ag/AgCl electrode at 11.26 ± 0.18 dB under static conditions. During wear testing, the electrode exhibited a high SNR of 32.60 ± 0.72 dB under static conditions and 30.27 ± 1.37 dB under dynamic conditions [176]. Shathi et al. designed graphene/PEDOT: PSS‐coated textile electrodes with low impedance and high conductivity for wearable ECG detection. The electrodes yielded high‐quality ECGs with very little distortion, achieving an SNR of 21.6 dB, comparable to that of clinical electrodes [177].

Ding Li et al. reported the first motion‐unrestricted dynamic electrocardiogram system, MU‐DCG, developed for imperceptible 12‐lead ECG monitoring during daily activities. This system is a new human‐machine fusion concept that integrates three critical components: imperceptible wearability, dynamic cardiac monitoring, and in situ disease analysis, serving as an “invisible doctor” for long‐term health monitoring. Performance was further validated through signal‐to‐noise ratio (SNR) analysis. The MU‐DCG system maintained a high average SNR (>23 dB) under both static and dynamic conditions. In contrast, the SNR of commercial Holter monitors decreased from ∼25 dB in static conditions to <17 dB during motion. In comparison, a conventional ECG pectoral girdle system exhibited lower SNRs (<17 dB static, <13 dB dynamic), demonstrating the MU‐DCG platform's superior dynamic noise resistance. In detail, Figure 2I(a) illustrates the schematic of the proposed system. MU‐DCG system consists of ultra‐thin (<50 µm), large‐area, skin‐conformal electrodes and leads fabricated on polymer fiber films, combined with helical interconnects for flexibility and breathability. The pressure‐activated, flexible skin socket is another important invention that provides a stable, soft connection between the on‐skin module and the off‐skin processing unit during dynamic movements. Compared with commercial Holter monitors, the MU‐DCG system could eliminate wires and cumbersome electrodes, thereby enabling imperceptible wearability, as highlighted in Figure 2I(b). Figure 2I(c) depicts the equivalent circuit model of the signal transmission path.

**FIGURE 2:**
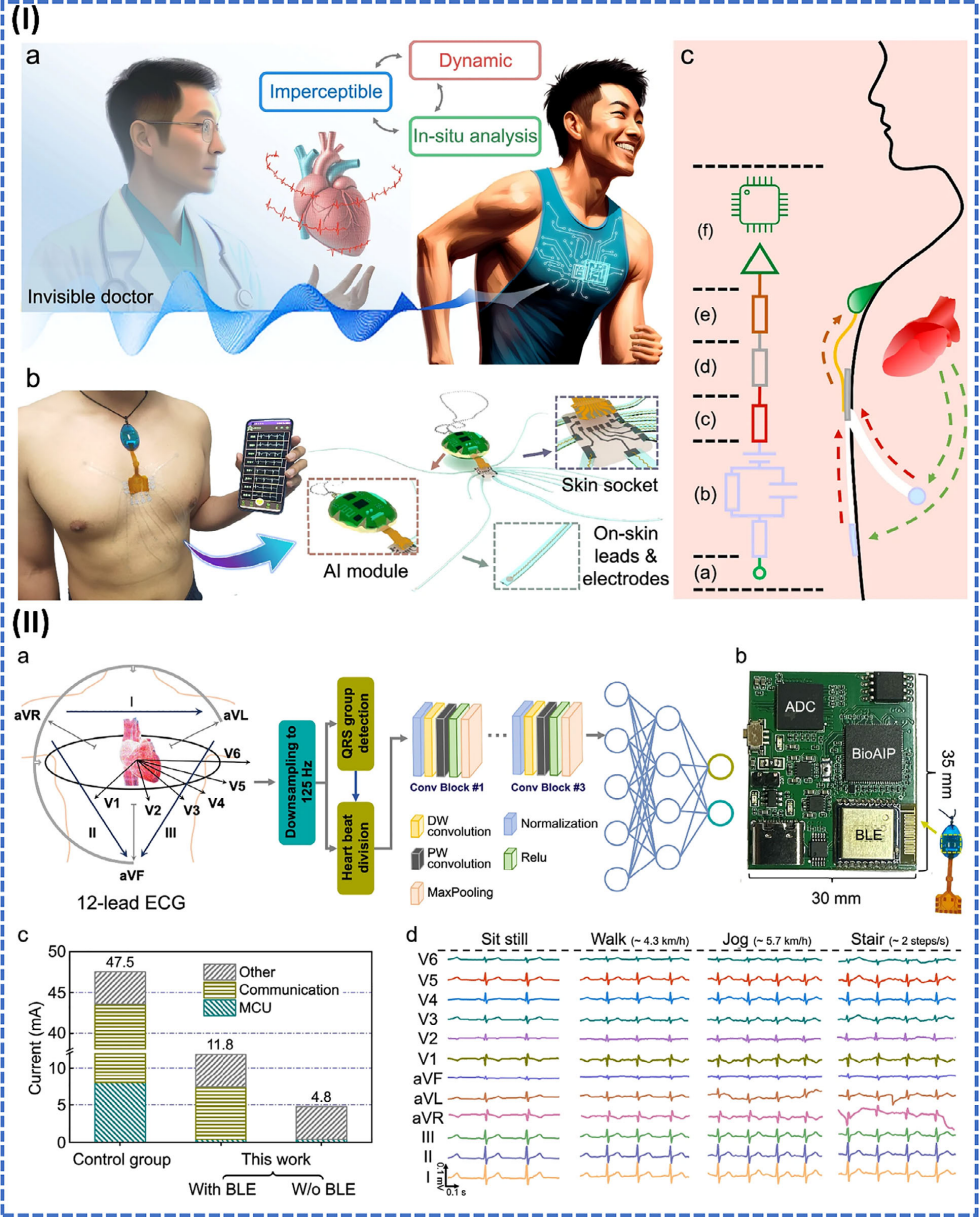
Smart Textile‐based ECG Electrode. (I) Architecture and design of the MU‐DCG System (a) Conceptual sketch of the MU‐DCG system operating as an imperceptible “invisible doctor” for continuous cardiac monitoring. (b) A subject wearing the MU‐DCG system comfortably, showing the elimination of bulky electrodes and wires compared to conventional Holter monitors. (c) The equivalent circuit model of the MU‐DCG signal transmission path includes the following optimised components to stabilise the signal: an integrated electrode‐lead design, an LM plug, a flexible skin socket, and an FPCB plug. (II). In Situ Analysis and Performance of the MU‐DCG System (a) Light‐Weight CNN Architecture for Myocardial Infarction Detection Based on 12 Lead ECG Data Collected By MU‐DCG. (b) Off‐skin processing module (30 mm × 35 mm) integrating BioAIP SoC, ADC, BLE, flash storage, and power management circuits for real‐time ECG analysis. (c) Power consumption is lower in MU‐DCG and MCU‐based systems, with extended battery life in BLE sleep mode. (d) Representative 12‐lead ECG signals captured during dynamic activities (sitting, walking, jogging, stair climbing), illustrating motion robustness with high signal fidelity. Reprinted under CC BY 4.0 license [178].

Beyond hardware design, the MU‐DCG system includes an off‐skin module that enables real‐time, low‐power ECG analysis. This module (30 mm × 35 mm) integrates a lightweight convolutional neural network (CNN) algorithm for myocardial infarction (MI) detection, which is deployed on this module as shown in Figure 2II(a), a self‐developed biomedical AI processing system‐on‐chip (BioAIP SoC), an analog‐to‐digital converter, Bluetooth Low Energy (BLE), flash storage, and power management circuits (Figure 2IIb). Performance evaluation shows that MU‐DCG achieves significantly lower power consumption than conventional MCU‐based systems, and the BLE sleep mode and independent operation can extend battery lifetime up to 10‐fold (Figure 2IIc). MU‐DCG achieved high‐quality 12‐lead ECG signals during various dynamic activities, including sitting, walking, jogging, and stair climbing (Figure 2IId). The reported MU‐DCG is a transformative platform for motion‐unrestricted cardiac monitoring in demanding scenarios, such as athletic performance assessment, aerospace missions, and emergency rescue [178].

A textile electrode for the slow release of moisture via an embroidery technique using superabsorbent polyacrylic fibers was fabricated by Liu et al. to create an optimal skin‐electrode microenvironment. The polyacrylic electrode was found to provide more stable signals than the polyester electrodes; for example, the ECG amplitude decreased by 17.1% after 10 h of moisture evaporation, whereas for polyester electrodes it decreased by 48.9%. In their studies, Liu et al. demonstrated dynamic ECG monitoring using a 5‐mm‐thick polyurethane sponge as an electrode base. The SNRs achieved were 25.39 dB and 30.09 dB for arm swinging and walking, respectively [179]. Sinha et al. created highly conductive graphene/PEDOT: PSS‐coated PET nonwoven textile electrodes with an ultra‐low resistance of 1.1 Ω/sq, thereby enabling dry‐skin ECG recording without hydrogel. Although the ECG waveform performance was demonstrated, no numerical SNR values were reported, suggesting the feasibility of conductive textile composite materials for wearable ECG recording applications [180].

Nigusse et al. designed a silver screen‐printed electrode based on a textile made of knitted cotton and polyester material for long‐term ECG recording. Both electrodes displayed ECG waveforms with SNR levels of 33.10 dB for cotton and 30.17 dB for polyester, while the Ag/AgCl electrodes had an SNR of 33.52 dB. Signal quality improved with higher contact pressure, and ECG recordings at 15 mmHg yielded results comparable to those of commercial electrodes [181].

Wang et al. examined a capacitive, non‐contact textile ECG system that captures the cardiac signal via the insulating properties of clothing material. The optimal ECG system exhibited an ECG SNR of 29.8 dB and a heart rate precision of 99.5% when compared to standard wet‐electrode measurements. In the experiment, the quality of the ECG signal depended significantly on the capacitance at the skin‐electrode contact, yielding a good ECG signal recording when the capacitance exceeded 85 pF [182].

To improve the electrode‐skin interface and reduce impedance, S. Lee et al. designed a 3D embroidered textile electrode comprising conductive yarns attached to a PDMS‐based substrate. This design led to an 80.6% decrease in impedance and a 5.42 dB improvement in ECG signal‐to‐noise ratio compared to regular flat textile electrodes. Moreover, the 3D structure exhibited consistent performance during body movement and correlated with the reference Ag/AgCl electrodes with a correlation coefficient of 0.893 [183].

Sliz et al. reported an approach to developing conformable electrodes for flexible electronics and smart textiles. In this regard, the author emphasises their involvement in reliable signal transmission and sensing in real‐world conditions. This study examines how mechanical elongation and washing cycles affect the structural integrity and electrical performance of R2R‐printed conductive electrodes of different shapes on various flexible substrates, manufactured using commercially available conductive pastes, as illustrated in Figure 3I. A comprehensive experimental framework was established using various electrode geometries and materials to investigate the mechanisms of breakage underlying electrical degradation. Physicochemical characterisations provided insights into nanoparticle morphology and microscale fracture processes, identifying promising particle shapes and sizes that enhance mechanical durability. These results highlight how to optimise electrode robustness to enable their integration into wearable systems for accurate ECG‐EEG monitoring via skin‐contact clothing. In this work, authors leverage scalable R2R printing and off‐the‐shelf materials to provide practical guidelines for fabricating robust, flexible electronic components, thereby enhancing the reliability and functionality of next‐generation wearable devices [184].

**FIGURE 3:**
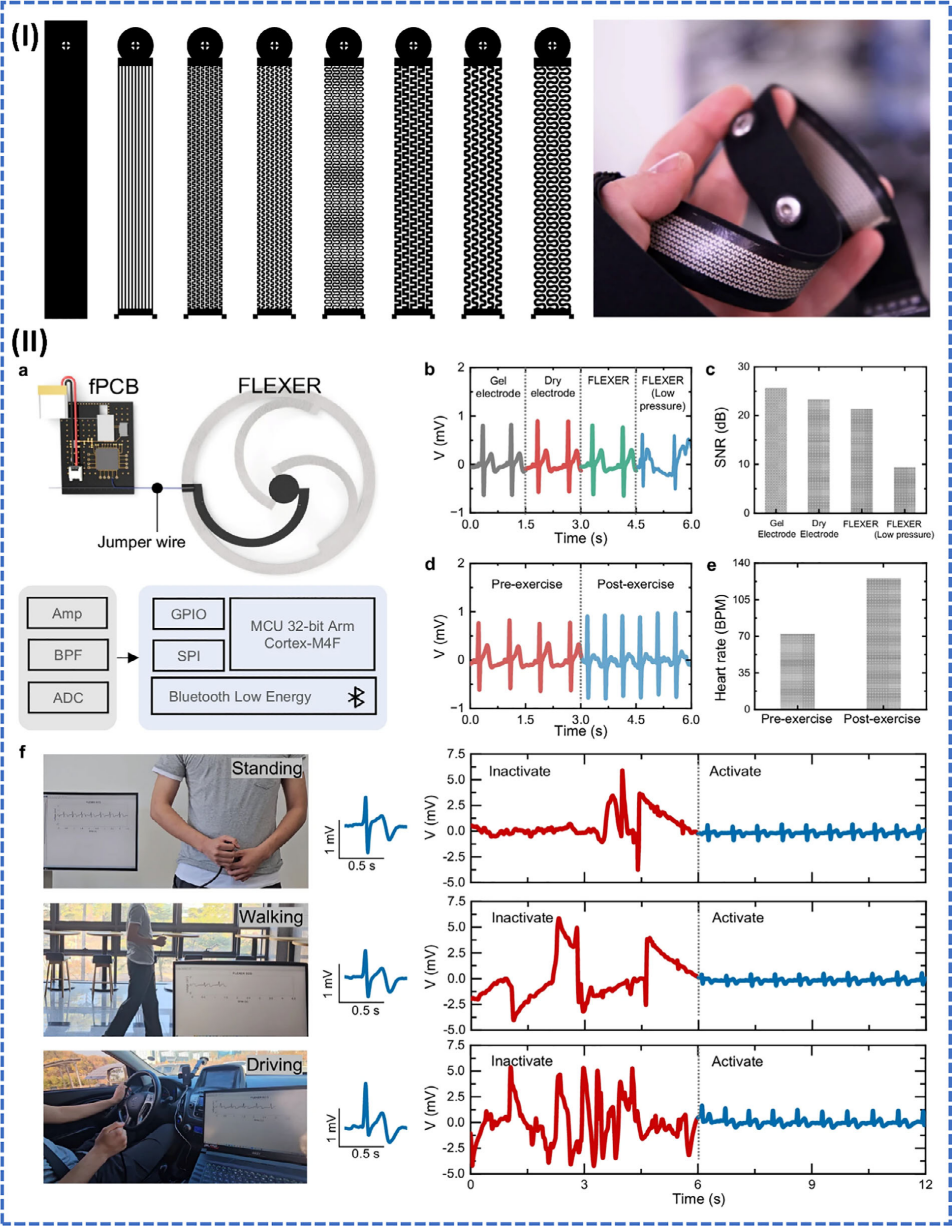
Textile‐based Electrodes for ECG monitoring (I) Various (115 × 20 mm) electrode shapes and visual implementation of electrodes in the fabric belt for ECG measurements, reprinted under CC BY 4.0 license [184]. (II) Flexer setup and performance in ECG acquisition. (a) Block diagram of the wireless monitoring setup (b) Performance comparison of ECG measurements acquired from FLEXER attached to clothing vs commercial gel‐type and dry Ag/AgCl electrodes. (c) SNR of FLEXER. (d) Flexer ECG acquisition performance before and after exercise. (e) The capability of FLEXER to detect changes in heart rate before and after exercise, at 72 and 125 BPM, respectively. (f) Real‐time ECG monitoring during dynamic activities, such as standing, walking, and driving, in both activated and non‐activated modes. reprinted under CC BY 4.0 license [185].

Lee et al. described an innovative solution to the discomfort and irritation often associated with conventional wearable biosignal sensors by developing a fabric‐based, emergent MXene electrode called FLEXER. The electrode combines the high electrical conductivity and low impedance of MXene with a shape‐morphing pneumatic structure inspired by Nesler's actuator design. They ensure stable skin contact without continuous pressure. Design parameters, including width, number of semicircular legs, anchoring frame radius, and pneumatic pressure, were optimised for diverse applications. To demonstrate the capabilities of FLEXER, they developed a real‐time wireless electrophysiological monitoring system in which the signals detected by FLEXER were routed to a flexible printed circuit board (fPCB), where they were subsequently amplified, filtered, converted using ADC, and wirelessly transmitted by Bluetooth Low Energy (BLE) for real‐time analysis.

Figure 3II shows some important features: Figure 3II(a) is the block diagram of the wireless monitoring setup; Figure 3II(b) compares ECG measurements acquired from FLEXER attached to clothing with commercial gel‐type and dry Ag/AgCl electrodes attached directly to the skin; in Figure 3II(c), the high SNR of FLEXER is due to its pneumatic structure, even while the sensor was separated from the skin by clothing. In contrast, poor SNR was observed under low air pressure. Figures 3II(d),(e) show the capability of FLEXER to detect changes in heart rate before and after exercise, at 72 and 125 BPM, respectively. Figure 3II(f) represents real‐time ECG monitoring during dynamic activities such as standing, walking, and driving. Wherein signal stabilisation was dramatic after FLEXER was activated. Of particular importance, the PQRST waveform fidelity achieved by FLEXER was comparable to that of commercial Ag/AgCl gel electrodes. At the same time, minimal discomfort was experienced because skin contact occurred only during measurement, thereby making it highly suitable for user‐friendly health monitoring over extended periods [185].

Furthermore, Laser‐induced hierarchical carbon nanofibers (LIHCNFs) were fabricated by Sharifuzzaman et al. from an economical PDFE/MXene precursor, which could serve as an alternative to PAN‐based carbon nanofibers. The porous, highly conductive carbon nanofibers with excellent mechanical stability and a sheet resistance of 4 Ω sq^−^^1^ were successfully synthesized via laser‐induced carbonization. Fabrication of a breathable electronic tattoo using these LIHCNF electrodes resulted in an electrode with a low skin‐electrode impedance value of 23.59 kΩ cm^2^ and high‐quality output signals with a signal‐to‐noise ratio (SNR) of 41 dB [186].

### 6.2 Smart Textile‐Based Pulse Monitoring

Recent progress in wearable pulse sensors has focused on developing sensors with high sensitivity, flexibility, and electromechanical durability for real‐time monitoring of health conditions [187]. Yang et al. have proposed a cost‐effective, customizable approach that uses machine embroidery to fabricate fully textile‐based sensors that can be easily integrated into smart garments. Yang et al. developed two configurations: a single‐layer satin block embroidered with conductive yarn, featuring high piezoresistivity, fast response (35 ms), quick recovery (16 ms), and operational durability over 5000 cycles for monitoring activities like plantar pressure and muscle contraction; and a double‐layer design allowing the integration of arrays, demonstrated by a 3×3 sensing array combined with a convolutional neural network realizing 98.5% accuracy in handwritten digit recognition, highlighting great potential for safe user authentication [188]. Similarly, large‐area all‐textile pressure‐sensor arrays on common fabric substrates were reported by Liu et al., who achieved high sensitivity (14.4 kPa^−^^1^), a low detection limit (2 Pa), a fast response time (∼24 ms), and low power consumption (<6 µW). These sensors, while maintaining mechanical stability under severe deformations, effectively recognized finger movements, hand gestures, acoustic vibrations, and real‐time pulse waves. They demonstrated that these sensors could be integrated into garments without compromising comfort, thereby advancing the promise of smart textiles [189].

Yoshimoto et al. proposed a pulse‐wave‐sensing textile that can continuously estimate blood pressure without stress during daily activities. Unlike most conventional ECG‐based fabric electrodes, PPG sensing in this design was incorporated into arrays of LEDs and phototransistors woven into conductive fibre circuits. The array configuration improved pulse‐detection stability during motion. This design demonstrated the feasibility of embedding pulse wave sensors into garments for continuous BP monitoring, combined with ECG electrodes [190]. Chen et al. employed a twisting force via a structural yarn design to reduce hysteresis without using elastic polymers. The pressure sensor demonstrated excellent performance, with a very low hysteresis of 5.3%, an ultrafast response and relaxation time of 2 ms, and a highly favorable recovery index, outperforming most reported textile sensors. The ability to monitor an epidermal pulse shows great promise for modernizing pulse palpation in traditional Chinese medicine and precision wearable health monitoring [191].

Meng et al. reported a low‐cost textile‐based sensor for comfortable, long‐term biomonitoring. The sensor has demonstrated a very high sensitivity of 3.88 V·kPa^−^^1^ and can accurately detect pulse waves even in weakened arterial signals in older individuals. Integrated into a wireless system, the TS transmits real‐time cardiovascular data to a mobile application, enabling continuous monitoring during sleep and facilitating the diagnosis of conditions such as obstructive sleep apnea‐hypopnea syndrome. Additionally, Figure 4I presents the structure and fabrication process of the TS. The sensor comprises a silver‐coated polyester base that serves as both an electrode and a triboelectric layer, together with an embossed, flower‐shaped textile for aesthetic purposes (Figure 4Ia). A commercial polyester fabric is first coated with silver to enhance the conductivity (Figure 4Ib‐c), as verified by SEM imaging (Figure 4Ie). A dielectric nylon yarn is sewn on a circular support (Figure 4Id).

**FIGURE 4:**
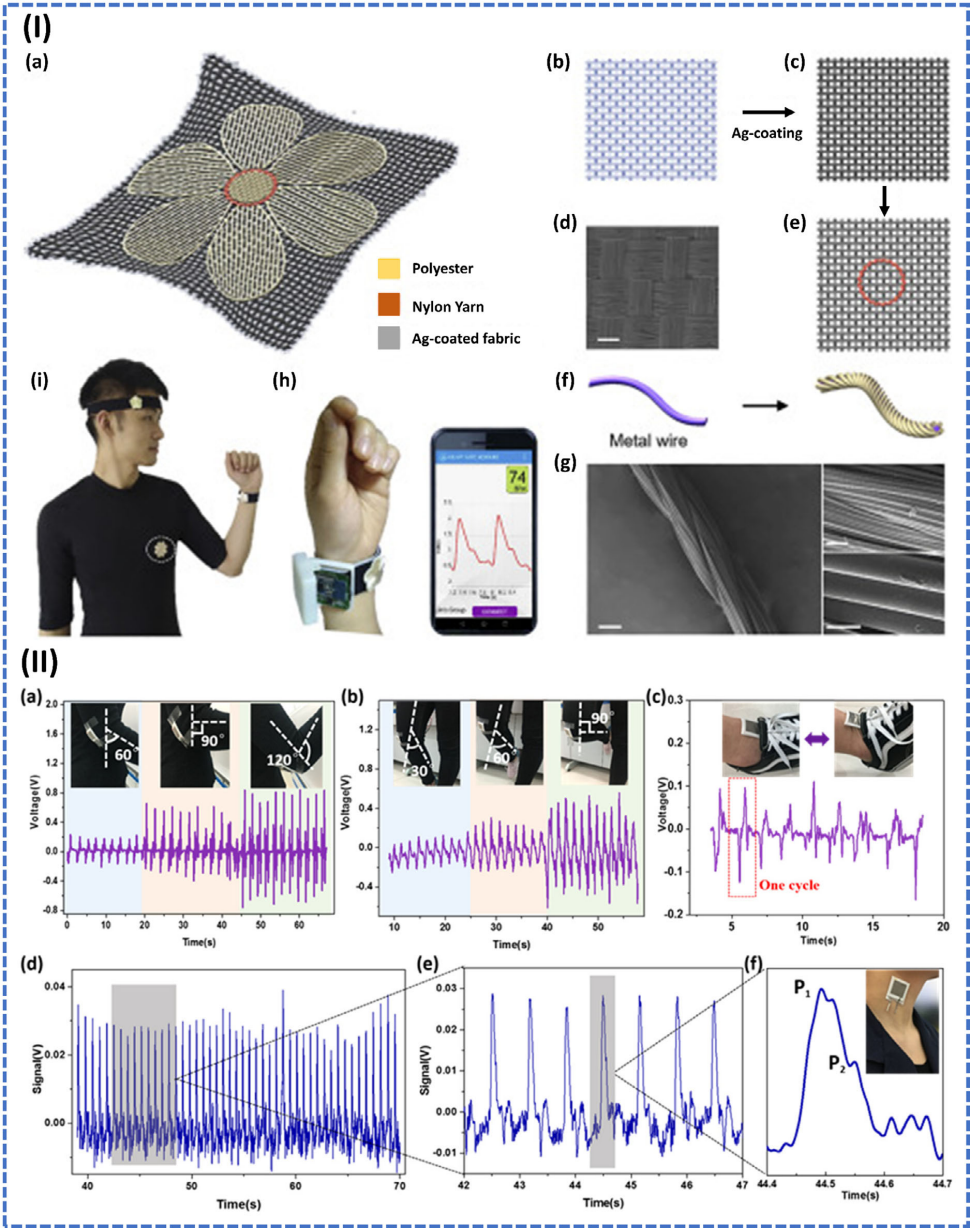
Smart Textile Application in Pulse Wave Monitoring. (I) Design and Integration of Textile Sensor System (a) Illustration of the fabricated textile‐based sensor (TS) featuring a two‐layer structure: a silver‐coated fabric serving as the base electrode and an embossed flower‐patterned textile forming the upper layer. (b–d) Stepwise schematic of the process used to create the silver‐coated electrode layer. (e) SEM image of the silver‐coated base layer (scale bar: 100 µm). (f‐g) Construction of the superstructure using polyester‐metal hybrid fibers, shown in (f) as a schematic and in (g) as SEM images at varying magnifications (scale bars: 100 µm, 20 µm, and 10 µm). (h) Photograph of a wearable TS‐based wireless health monitoring system displaying cardiovascular data on a smartphone via wireless transmission. (i) A photograph demonstrating the versatility of the system, which can be worn on various body parts for health monitoring. Reprinted with copyright permission of [192] 2019 Elsevier Inc. (II). Applications of Pressure Sensor Textile in Motion and Pulse Detection (a–c) Detection of joint movements at varying angles for the elbow, knee, and ankle. (d, e) Real‐time monitoring of carotid artery pulse waves using the textile sensor. (f) Enlarged view of the pulse waveform showing two peaks: P1 (pulse pressure, representing systolic–diastolic difference) and P2 (reflected wave pressure from the hand). Reprinted with copyright permission of [193] 2020, American Chemical Society.

In contrast, a polyester‐metal hybrid fiber was obtained by twisting a polyester yarn around a metal wire, thereby providing an additional triboelectric layer (Figure 4If‐g). These conductive fibres were stitched into decorative floral patterns and embossed onto the silver‐coated textile. The TS can be integrated into various garments and devices at different body sites, including the wrist, forehead, and chest (Figure 4Ii), and can be connected to a wireless monitoring system to display real‐time data on smartphones (Figure 4Ih). This work presents a high‐sensitivity, aesthetically tunable textile sensor, combined with a wireless system, for continuous cardiovascular monitoring, providing a practical solution for personalised health assessment [192].

Lou et al. designed a triboelectric all‐fiber pressure sensor fabric based on a facile electrospinning process, combining a polyvinylidene fluoride/Ag nanowire nanofibrous membrane (NFM), an ethyl cellulose NFM, and two conductive fabric layers. Lou et al. show that a hierarchically rough nanofiber surface dramatically improves triboelectric properties, yielding high sensitivity of 1.67 V/kPa over 0 to 3 kPa and 0.20 V/kPa over 3 to 32 kPa. Lou et al. developed a sensor that exhibits excellent mechanical durability, withstanding 7200 cycles, and superior shape adaptability. Moreover, in practical applications, Lou et al. developed a sensor that demonstrates excellent conformability to any part of the human body and the ability to sense dynamic movements, as shown in Figure 4IIa‐c. For elbow postures at 60°, 90°, and 120°, the output voltage increased from 0.23 to 0.65 V, then to 0.84 V, as pressure increased with increasing angle. For the bent‐knee postures at 30°, 60°, and 90°, the output voltage increased from 0.16 to 0.30 V, then to 0.55 V. Moreover, ankle movements produced periodic, rhythmic changes in the output voltage, confirming the system's ability to accurately detect dynamic human body movements.

Most importantly, (Figure 4IId‐f) confirms the sensor's capability to accurately measure the heartbeat in real‐time when positioned near the carotid artery, and it was able to capture seven peaks in 5 s, corresponding to an average of 84 beats per minute, well within the normal limits. The magnified graph clearly shows two peaks: P1, corresponding to the pulse pressure (systolic and diastolic pressures), and P2, corresponding to the reflected‐wave pressure. The augmentation index (AIx = P2/P1) was estimated at 47.08%, similar to that of a healthy individual under 35 years of age [193].

Recent advancements in nanofiber‐based and piezoelectric sensors have significantly improved wearable pulse and cardiovascular monitoring systems. Zou et al. introduced a nanofiber strain sensor combined with a novel blind source separation framework, using noise‐assisted multivariate empirical mode decomposition (NAMEMD) and multiset canonical correlation analysis (MCCA) to accurately extract heartbeat signals from noisy environments. The proposed method achieved an average absolute correlation coefficient of 0.902, outperforming existing approaches and preserving signal morphology during various tasks [194]. Moghadam et al. prepared a PVDF nanofibrous membrane incorporating a zirconium‐based MOF. They demonstrated a 3.4‐fold increase in constant piezoelectric response, with a peak‐to‐peak voltage of 600 mV under an applied force of 5 N. The sensor showed higher sensitivity (0.118 V/N) and output voltage of 568 ± 76 mV for wrist pulse monitoring due to enhanced crystallinity and β‐phase content [195].

Kang et al. reported a peapod‐inspired design with ZnSnO_3_ anchored on surface‐modified carbon nanotubes within P(VDF‐TrFE) nanofibers, featuring excellent piezoelectric performance, including a high output power of 97.5 V and 1.16 µA, and imperceptible pulse detection in posterior tibial arteries, promising applications in energy harvesting and sensing [196]. Md Nayeem et al. developed an ultrasensitive all‐nanofiber mechanoacoustic sensor for long‐term monitoring in cardiovascular diseases. It achieves a very high sensitivity of 10,050.6 mV·Pa^−^^1^ at the low‐frequency range (<500 Hz), low weight (5 mg), and mechanical robustness up to over 1,000 bending cycles, and allows continuous heart signal measurement for up to 10 h with an SNR of 40.9 dB [197].

N. Das et al. present a single‐electrode triboelectric nanogenerator for underwater arterial pulse monitoring and wireless transmission. The developed STENG used Ni‐MOF/PVDF composite nanofibers and nylon 66 nanofibers encapsulated by PDMS for waterproofing. The STENG exhibited a Voc of 45 V and an Isc of 0.77 µA upon finger tapping, performed well in both saline and muddy water, and sensed joint movements, thereby establishing its potential for underwater, real‐life health monitoring [198].

Lou et al. designed a triboelectric sensor composed of core‐shell fibres prepared using a speed‐controlling motor, with nylon and PTFE as the positive and negative triboelectric materials, respectively, and a helical stainless‐steel fibre serving as the inner electrode. The cloth was woven on a wooden weaving machine, with a warp density of 10 lines/4 cm and a weft density of 28–32 lines/4 cm, to embed the electrode materials in the fibres safely and prevent leakage of electric current. Based on Figure 5I(a), the preparation process consisted of winding the stainless‐steel fibres with PTFE and nylon threads to get two kinds of weft fibres, and the remaining warp fibres were made only from PTFE and nylon threads. The cloth exhibited high sensitivity values of 1.33 V/kPa (1.95 ∼ 3.13kPa) and 0.32 V/kPa (3.20 ∼ 4.61kPa), along with excellent mechanical robustness, even after undergoing 4200 cycles of mechanical testing and a 4‐h water wash test. By taking advantage of the fabric's properties, including breathability, flexibility, lightness, and dyeability, the sensor could be made in various colors, such as light blue, dark blue, and yellow (Figure 5Ib). Moreover, the fabric sensors consisted of four versions: PP (PTFE warp and weft), PN (PTFE weft and nylon warp), NN (nylon warp and weft), and NP (nylon weft and PTFE warp) (Figure 5Ic). Since the electronegative PTFE and electropositive nylon formed the best combination, the resulting PP and PN fabrics served as the negative triboelectric materials in the TENG sensor. In contrast, the NN and NP fabrics served as the positive part. The sensor cloth detected dynamic human movements at the joints (hand, elbow, knee, and underarm regions) and real‐time pulse signals, thereby representing a promising approach to intelligent fabrics and personalised medicine [199].

**FIGURE 5:**
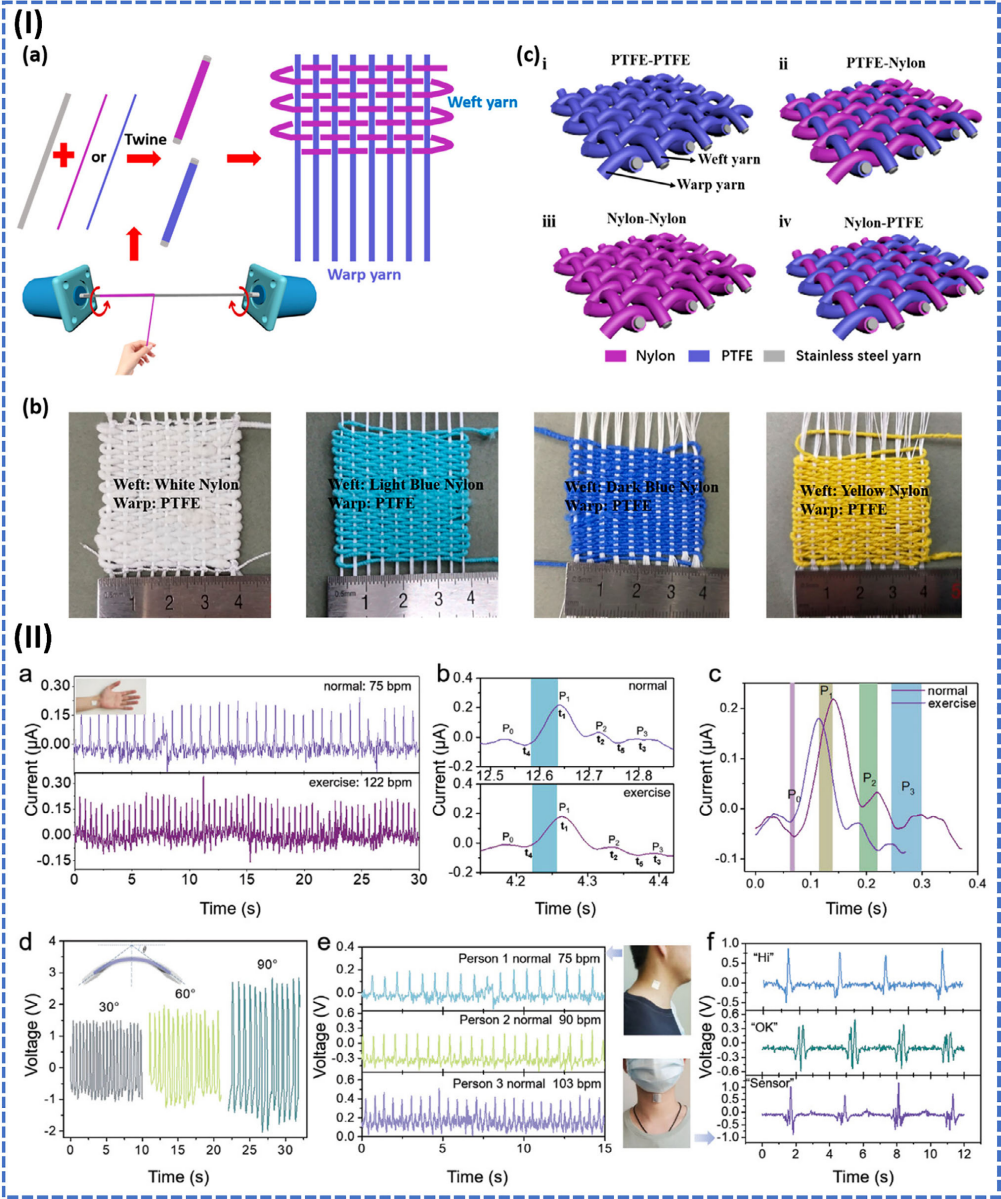
Smart Textile Application in Pulse Wave Monitoring. (I) Structural design and development process of the pressure sensor textile. (a) Schematic illustration of the development process of the core‐shell yarns. (b) Dyeability of the pressure sensor textile. (c) Various weave designs for fabrics made from core‐shell yarns. Reprinted with copyright permission of [199] 2020 American Chemical Society (II) Performance of the prepared muscle fiber‐inspired (MFP) textile to monitor physiologic functions. a) Real‐time current output waveform of pulse for static status and dynamic status following exercise. Inset: Photograph of the device put on the wrist of a volunteer. b) Expanded versions of a cycle of electrical signals having three typical peaks (P1, P2, and P3) for static status and dynamic status following exercise. c) Single pulse wave analysis for static status and dynamic status following exercise. d) Real‐time output voltage waveform of finger bending at various angles. e) Pulse waveforms of various volunteers with the same positioning of the fabricated MFP textile on their respective necks. f) Dynamic output waveform for spontaneous voice recognition while pronouncing various words. Reprinted with copyright permission of [200] 2021 Wiley‐VCH GmbH.

Su et al. designed a muscle fibre‐inspired (MFP) piezoelectric nonwoven textile by incorporating polydopamine (PDA) into barium titanate/polyvinylidene fluoride (BTO/PVDF) nanofibers, thereby enhancing interfacial binding, mechanical properties, and piezoelectric performance, as verified by experiments and phase‐field simulations. The resulting flexible, ultralight textile exhibited high sensitivity (3.95 V/N) and long‐term electromechanical durability (<3% loss after 7,400 cycles), making it suitable for pulse monitoring, gesture tracking, and voice recognition. In practical application, as highlighted in Figure 5IIa, the textile mounted above the radial artery accurately monitored changes in the wrist pulse waveform, with a heart rate of approximately 75 beats per minute that increased to 122 beats per minute following exercise. Figure 5 IIb shows three characteristic peaks: P1 (percussion), P2 (tidal), and P3 (diastolic) components, along with augmentation indexes (AIx = P2/P1) of 0.416 and 0.459 at rest and following exercise, respectively, clearly demonstrating fast‐response and cardiovascular function‐testing capabilities. Figure 5IIc shows the pulse waveforms measured in stationary and excited modes, with periods of 39 ms and 27 ms, respectively. For gesture tracking, Figure 5IId shows the proportional voltage outputs for finger angles ranging from 30° to 90°. Figure 5IIe illustrates the carotid artery pulse waveforms measured in three human subjects, clearly identifying characteristic points P1‐P2 according to the persons´ differing health conditions. The last application in Figure 5IIf demonstrates the capability of the sensor for voice control to give the corresponding voltage signals to the words “Hi,” “OK,” and “sensor,” making it clear that the sensor has the potential to function in human‐machine interfaces based on voice operations [200].

### 6.3 Smart Textile‐Based Hemodynamic Monitoring

Hemodynamics, the study of the mechanics of blood circulation, can significantly contribute to cardiovascular health. However, it still poses various challenges due to its branched nature and complex viscosity profiles [201]. Traditionally, optical sensors have been preferred for monitoring hemodynamic characteristics in human subjects; however, the complex vascular anatomy makes this very challenging [202]. To address these challenges, Qiu et al. have suggested an alignment‐free electrophysiological sensor sleeve based on textile technology: an alignment‐free electrophysiological sensing sleeve (TAESS). Qiu et al. integrate impedance plethysmography and electrocardiography to simultaneously monitor blood pressure, cardiac output, systemic vascular resistance, and heart rate. Qiu et al. developed a device that uses automated knitting with silver‐based conductive yarns. It boasts very high breathability, a stretch ratio of more than 45 percent, and stability against thermal changes, even after exercise.

Additionally, Qiu et al. developed a device that exhibits very high signal resolution compared to existing electrodes, with an error value of 7.05 mmHg, and accurately forecasts systolic and diastolic values. Additionally, Figure 6Ia shows that the device developed by Qiu et al. remains position‐independent and operates with an isotropic rotating sleeve at 0°, 90°, and ‐90°. Additionally, it clearly shows the variability of blood flow and physiological signals across these three positions (Figure 6Ib). So, in impedance plethysmography, Qiu et al. developed a device that produced very clear and precise signals related to R.R. intervals and dz/dt. On the other hand, traditional PPG biosensors with very low optical penetration exhibit almost no signal even after removing vascular alignment, as shown in Figure 6Ic‐f [203].

**FIGURE 6:**
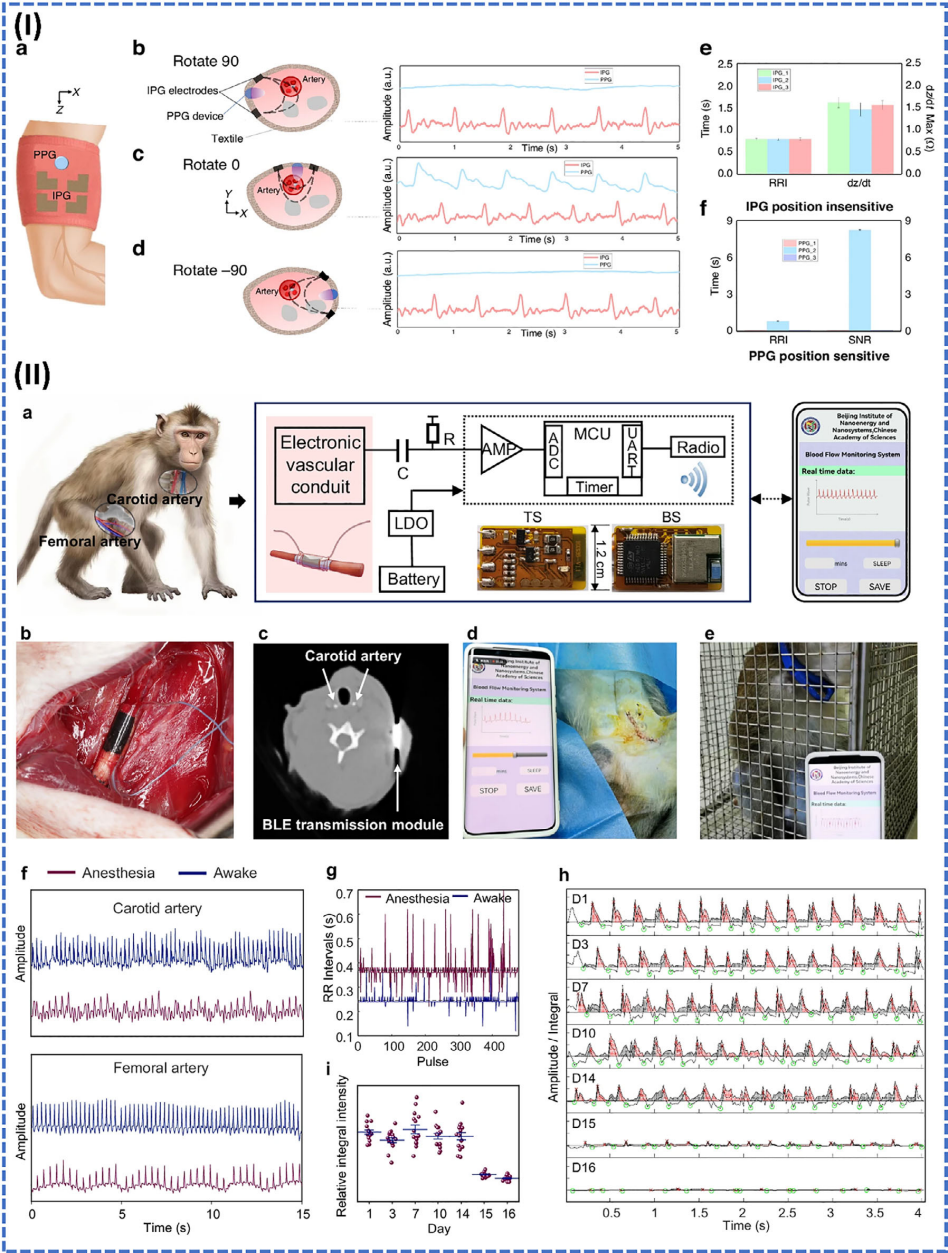
Application of Nanofibers and Textiles in the Hemodynamic Monitoring. (I) Alignment‐independent performance of TAESS for wearable hemodynamic monitoring. (a) Sensor placement on the arm. Radial rotation of the IPG electrode by (b) 90°, (c) 0°, and (d) −90°, with comparison of the PPG device influence on blood flow signal capture. (e) Quantified RRI and dz/dt across rotations. (f) RRI and PPG signal‐to‐noise ratio under varying sensor orientations, reprinted from [203], under CC BY license. (II) demonstrates real‐time wireless hemodynamic monitoring in cynomolgus monkeys using the electronic vascular conduit. (a) Block diagram of the BLE‐enabled transmission module, including amplification, signal processing, and wireless communication; inset shows optical images of the module. (b–c) Experimental setup and CT image post‐implantation. (d–e) Real‐time pulse wave display during anaesthesia and awake states, with (f) comparative waveforms from carotid and femoral arteries. (g) Statistical analysis of RR intervals and (h) continuous wireless monitoring over 14 days, during which anticoagulation was stopped on day 14 to induce thrombosis. (i) Relative pulse wave integrals across different days, confirming stable performance. Data are shown as mean ± SD (n = 15 pulses, 3 experiments) and reprinted from [206], under CC BY license.

Sempionatto et al. developed a stretchable, conformal wearable platform capable of simultaneous monitoring of blood pressure (BP), heart rate (HR), and multiple biochemical markers (glucose, lactate, caffeine, alcohol) for comprehensive health tracking. The system integrates ultrasonic transducers for hemodynamic measurements and electrochemical sensors for biomarker detection. Through optimized material selection, structural design, and fabrication strategies, including solvent soldering of rigid piezoelectric PZT ultrasound components to soft polymer composites, the device achieves mechanical robustness and eliminates sensor crosstalk. This hybrid epidermal sensor enables real‐time cardiovascular and metabolic monitoring by combining ultrasonic pulse‐echo sensing with iontophoretic extraction of interstitial fluid and stimulation of the sweat glands. On‐body trials under various physiological conditions (exercise, food, alcohol, caffeine intake) demonstrated strong correlations between metabolic and hemodynamic responses. The use of SEBS‐based stretchable substrates enabled rapid, reliable assembly of the multimodal sensor, addressing integration challenges and supporting remote, telemetric health monitoring in post‐pandemic contexts [204]. Rosalia et al. developed a biomimetic soft‐robotic aortic sleeve to emulate the hemodynamic conditions associated with pressure overload in AS. Such an active, highly tunable sleeve enables patient‐specific modelling by altering actuation patterns or sleeve geometry, providing a high‐fidelity in vivo platform for disease simulation. Beyond AS, this technology holds great promise for modelling various pathologies, including vascular disorders such as carotid stenosis, peripheral arterial disease, and aortic coarctation; pulmonary valve stenosis; and functional obstructions of the urinary, gastrointestinal, and airway systems. Such personalised models are very useful for gaining insight into complex pathophysiology and support translational research, thereby enabling innovation in therapeutic strategies and medical device development [205].

Liu et al. reported an electronic vascular conduit that incorporates flexible electronics into bionic vascular grafts to monitor hemodynamics in real time over the long term and to detect hemodostenosis and thrombosis as early as possible after bypass surgery. Liu et al.’s system consisted of a triboelectric‐based self‐powered sensor and a wireless transmission module, enabling continuous monitoring for up to 3 months. Additionally, Figure 6II illustrates the wireless monitoring architecture and in vivo performance of Liu et al. device: (a) block diagram of the BLE‐enabled module for signal acquisition, amplification, filtering, and transmission to a smartphone app; (b–c) surgical implantation of the conduit and embedded electronics in cynomolgus monkeys; (d–e) real‐time wireless display of pulse wave signals on the app; (f–h) dynamic changes in pulse rate and amplitude between anesthesia (174 bpm) and awake states (252 bpm); and (i) stable signal output over 14 days post implantation. Liu et al.’s platform demonstrates good sensitivity and reliability for postoperative monitoring. It is thus envisioned to prevent graft failure and improve patient outcomes in the management of CAD and PAD [206].

Boutry et al. presented a wireless, battery‐free cuff‐type sensor for intraoperative monitoring of arterial blood flow. The device features a thin and flexible design, enabling it to be placed on arteries of various sizes. The use of radio‐frequency coupling makes it suitable for wireless operation. Manufactured exclusively from biodegradable materials, the sensor is naturally resorbed within months with no surgical removal required. Performance validation was carried out both in vitro, using an artificial artery model, and in vivo in rats, demonstrating excellent biocompatibility and accurate pulse‐monitoring performance in both wired and wireless configurations [207]. Si et al. created a textile sensor with a multi‐stage structure for acquiring ballistocardiogram signals, enabling unconstrained blood pressure measurement. The textile sensor, made from a nylon substrate with micro‐cilia and support layers, demonstrated a high sensitivity of 0.533 V/kPa over a static pressure range of 0–7 kPa, equivalent to body weight. BCG signals obtained from the sensor were consistent with cardiac oscillations measured with a laser vibrometer with 0.5 pm resolution. From precise BCG signals, a back‐propagation neural network model for cuffless blood pressure measurement was developed. It ensured mean absolute errors within AAMI Class II standards at 3.90 ± 4.79 mmHg and 4.62 ± 6.00 mmHg for diastolic and systolic pressures, respectively [208]. Li et al. introduced a wearable ultrasound sensor for capturing blood pressure waveforms. Based on an S‐shaped stretchable island‐bridge electrode and hot‐pressed transfer bonding to a fabric substrate, a 4×5 sensor array can detect deep‐lying arterial vessels with a high axial resolution of 330 µm. Catering to comfort and air‐permeability factors, it exhibits high stretchability with 35.6% elongation at break. It supports user comfort while enabling observation of hemodynamic patterns [209].

Ma et al. introduced an all‐in‐one piezoelectric vascular graft with high sensitivity, suitable for precise hemodynamic monitoring. The piezoelectric vascular graft contains a PVDF nanofiber mat with patterned silver nanowire electrodes, which are encapsulated in two layers of PCL nanofibers. Based on its optimal structure with ≈ 950 nm and 250 nm diameter PVDF and PCL fibers, respectively, it shows excellent biocompatibility and acceptable mechanical properties, closing the gap with natural vessels. Ma et al. utilised the piezoelectric properties of PVDF, demonstrating high sensitivity (11 mV/kPa) and cyclic life (>50,000 times). Ma et al.’s device can precisely measure the pressure and vascular status of an artificial artery model. Moreover, excellent biocompatibility, including nontoxicity, hemocompatibility (hemolysis ratio < 1%), and histocompatibility, demonstrates that this piezoelectric vascular graft offers considerable advantages over traditional vascular materials [212].

Tang et al. have proposed an implantable, unconstrained vascular electronic system for wireless, real‐time hemodynamic monitoring during arterial growth. Tang et al.’s system integrates a piezoelectric sensor composed of circumferentially aligned PVDF nanofibers and a biomimetic, “growable” sheath inspired by leaf morphology. This elastic PU nanofiber sheath adapts to the dynamically growing artery at appropriate speeds, ensuring conformal attachment without constriction or loss of sensor sensitivity. The device is designed for continuous monitoring of blood pressure, heart rate, and respiratory waves, and provides wireless data transmission to a mobile terminal without batteries, thereby reducing the risk of infection. Additionally, Figure 7I represents the Tang et al. device concept: (a) schematic of the implantable system for dynamic monitoring; (b) bioinspired sheath structure for adaptive growth; and (c) long‐term sensor stability in rats and rabbits, as arteries grow from infancy to adulthood. Tang et al.’s approach offers a minimally invasive, versatile strategy for implantable electronics that may address significant challenges in long‐term vascular monitoring and postoperative care [210].

**FIGURE 7:**
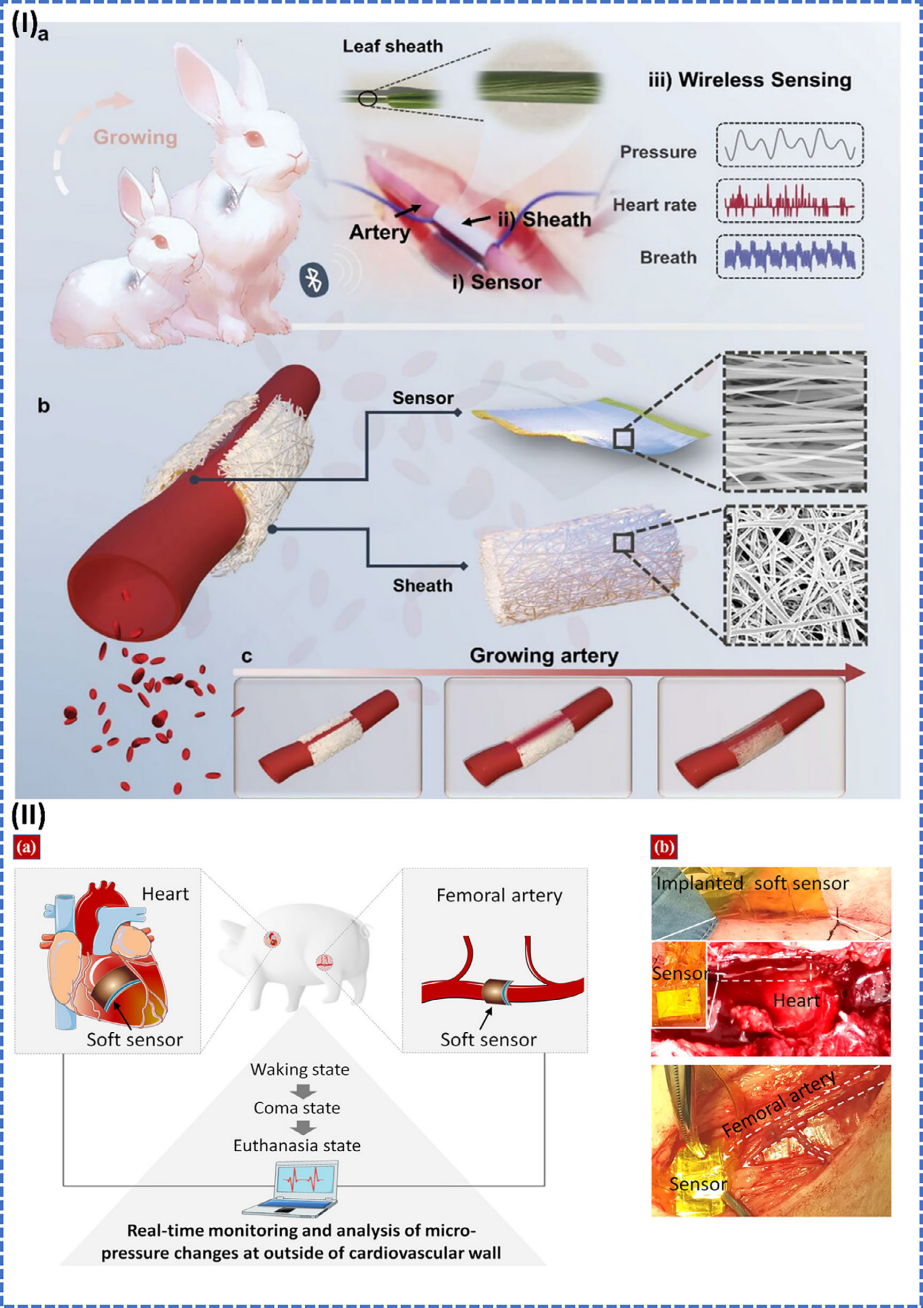
Application of Nanofibers and Textiles in the Hemodynamic Monitoring. (I) Overview of the unconstrained vascular electronic system: (a) schematic of the implantable setup, comprising (i) a flexible piezoelectric sensor for real‐time hemodynamic monitoring, (ii) a bioinspired growable sheath for secure immobilization around the artery, and (iii) a wireless data transmission module; (b) conceptual illustration of the sensing process, showing the sensor conformally adapted to the artery with the sheath; and (c) diagram depicting the sheath‐based immobilization strategy. This design ensures long‐term stability, adaptability to arterial growth, and minimally invasive wireless monitoring, Reprinted with copyright permission of [210], 2023 Wiley‐VCH GmbH. (II) Illustration of real‐time in vivo monitoring of micropressure changes on cardiovascular walls: (a) schematic showing sensor implantation for pressure recording; (b) surgical images of soft sensors attached to the heart and femoral artery. Reprinted with copyright permission of [211], 2019 American Chemical Society.

Li et al. proposed a structural design strategy for core/shell PVDF/HHE organic piezoelectric nanofibers with spatially uniaxial β‐phase orientation, offering significantly improved piezoelectric performance, fatigue resistance, and biocompatibility. Li et al. developed OPN‐based soft sensors implanted in pigs to monitor micro‐pressure changes in the cardiovascular walls. The sensor exhibited very high sensitivity and accuracy, enabling real‐time detection of changes in cardiovascular elasticity and early signs of atrioventricular block or thrombus formation, which are critical for diagnosing and treating thrombosis and atherosclerosis. Figure 7II presents the in vivo test of the coaxial piezoelectric nanofibers: (a–b) sensors implanted on the heart and femoral artery, reflecting the high performance of the sensor. These facts further confirm the sensor's ability to detect slight biomechanical variations and provide a basis for early diagnosis and prevention of vascular disease by addressing the limitations of conventional imaging techniques [211].

### 6.4 Smart Textile‐based Cardiovascular Tissue Engineering

There have been promising developments in textile scaffolds for use with cardiovascular tissue engineering. Jiang et al. introduced a wet electrospinning method to produce polymer/CNT composite yarns with improved mechanical properties, biodegradability, and cell alignment. The yarns were knitted into vascular scaffolds and combined with targeted textile and hydrogel materials to better simulate the elasticity and cellular proliferation of natural tissues [213]. Derya et al. introduced pile‐loop knitted PET materials as 3D scaffolds for myocardial tissues, featuring tensile properties (40–167kPa) and interspersed porosities (100‐400µm) that are qualitatively similar to those of cardiac tissues, thereby facilitating biocompatibility and regeneration [214]. Xie et al. introduced a two‐layer arterial prosthetic comprising weft‐knitted PLAs intertwined with electrospun PLCL and collagen and elastin coatings, which simulated extracellular tissues with enhanced biocompatibility, mechanical stability, and viability [215]. Eslami et al. introduced a combination of electrospun PGS‐PCL scaffolds and methacrylated hydrogel composites, demonstrating improved retention of tensile strength and biocellular distribution for bioregenerative valve functions [216]. Mohapatra et al. introduced textile‐reinfused biohybrid vascular scaffolding comprising a PVDF mesh and electrospun PLGA, targeting aneurysm decompression and bioreactor functionality [217].

Recently, novel textile‐based cardiac tissue engineering approaches have sought to combine nanofiber scaffolds with sophisticated bioelectronic/biomimetic systems that can efficiently emulate the anisotropic structure and microenvironment of the natural myocardium. Fan et al. added a supramolecular approach to replicate cell‐mediated remodelling of the ECM for mesenchymal stem cell (MSC)‐based therapy. They engineered a D‐phosphopeptide (Nap‐pD‐E7), which assembles via enzyme‐catalysed self‐assembly, producing a dynamic fibrillary matrix that supports MSC spheroid formation and paracrine effects. Specifically, there is an initial oligomerisation of phosphopeptides into nanoparticles that support MSC attachment, which is later adjusted by partial dephosphorylation via intracellular phosphatases, resulting in the generation of a fibrillary structure and thereby providing a responsive microenvironment as illustrated in Figure 8I. When MSC spheroids were cotransplanted with the self‐assembled hydrogel, marked improvement in MI murine hearts was evident. This approach demonstrates the potential of supramolecular systems to noninvasively manipulate stem cell behaviour, making them useful for future applications in ischemic heart disease [218].

**FIGURE 8:**
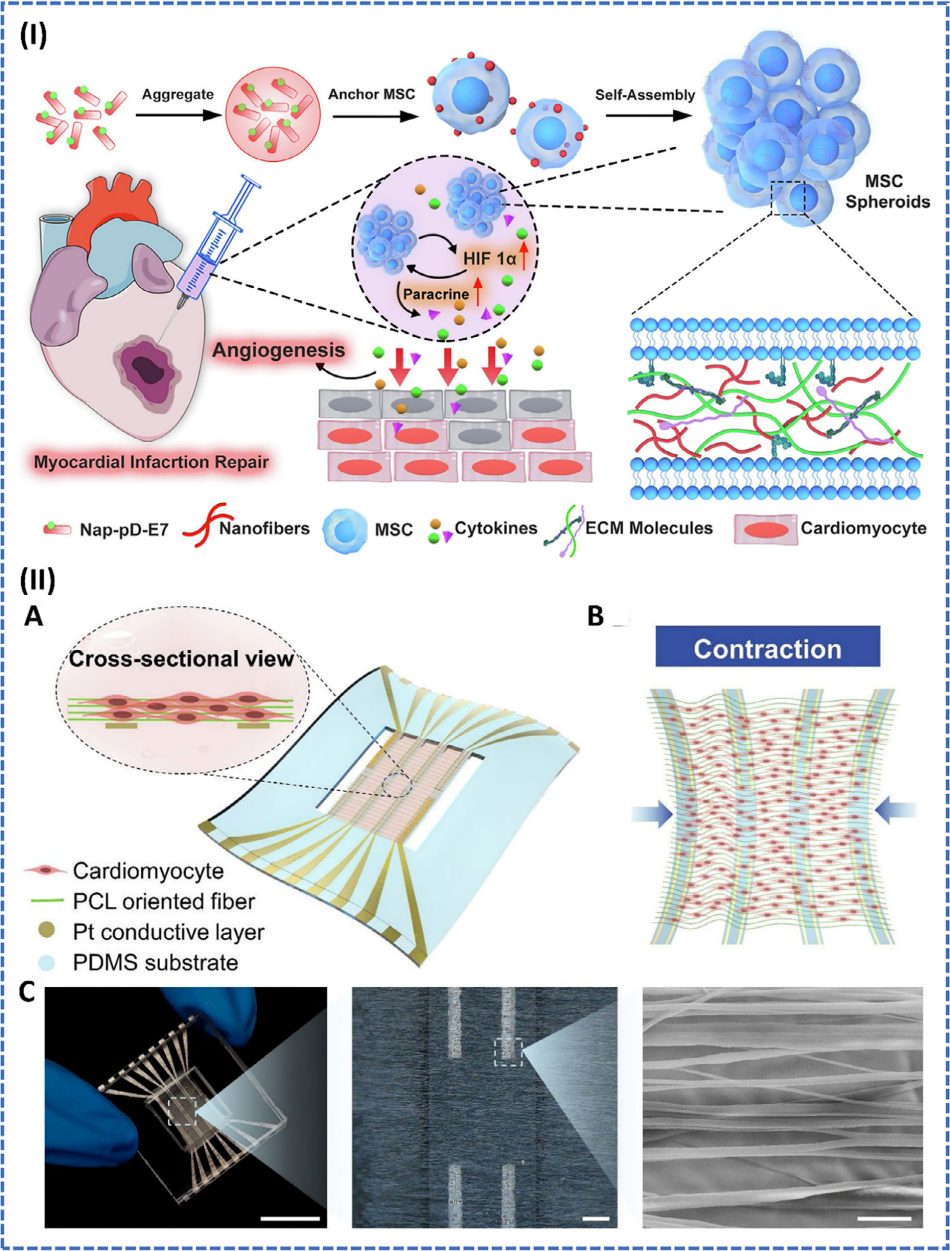
Application of Textiles and Nanofibers in Tissue Engineering Applications. (I) Schematic representation of the process by which Nap‐pD‐E7 phosphopeptides induce the formation of mesenchymal stem cell (MSC) spheroids, followed by their encapsulation within a Nap‐pD‐E7 hydrogel for co‐transplantation. This approach enhances MSC paracrine signalling and improves therapeutic efficacy in the treatment of myocardial infarction (MI). Reprinted with copyright permission of [218], 2022 Elsevier B.V. (II) Overview of the structure and fabrication of the beam‐based microelectrode array (BMEA). (A) Schematic representation of the BMEA design. (B) Illustration showing deformation of flexible beams during cardiomyocyte contraction. (C) Physical view, optical microscopy, and SEM images of the BMEA, with scale bars of 10 mm, 200 µm, and 5 µm, respectively. Reprinted with copyright permission of [219], 2025 Acta Materialia Inc. Published by Elsevier Inc.ii.

Zhang et al. introduced a flexible PDMS beam microelectrode array (BMEA) that is combined with a PCL nanofiber scaffold to electrophysiologically monitor the development of aligned cardiac tissue in a 3‐D microculture setting. The BMEA integrates PDMS beams with a microelectrode array comprising microscale platinum electrodes (Figure 8II), along with a top‐mounted, oriented PCL nanofiber scaffold that seeds human‐induced pluripotent stem cell‐derived cardiomyocytes (hiPSC‐CMs) on a low‐modulus, anisotropic growth surface (Figure 8IIA). The PDMS beam structure is deformable due to cardiomyocyte contraction (Figure 8IIB), thereby dynamically facilitating the propagation of electrophysiological signals. In particular, the BMEA has a microelectrode array with 14 recording electrodes (100 µm × 100 µm) and two reference electrodes (500 µm × 100 µm) (Figure 8IIC). Continuous electrophysiological analysis has documented improved cardiac development, including increased cardiomyocyte alignment, increased expression of mature cell markers, and increased electrophysiological conduction velocity, thereby verifying the utility of BMEAs for pharmacological analysis and electrical cardiac modelling [219].

Kobuszewska et al. reported a microfluidic platform that incorporates poly‐L‐lactic acid (PLLA) and polyurethane (PU) nanofiber scaffolds fabricated by solution blow spinning (SBS). The use of these aligned fibers promoted parallel alignment and viability of rat cardiomyoblasts (H9C2) and human cardiomyocytes (HCM), while the platform efficiently replicated hypoxic conditions using FCCP, with ATP depletion quantified [220]. In a similar application, Streeter et al. described electrospun PCL nanofiber scaffolds that pair with c‐kit+ cardiac progenitor cells (CPCs), aiming to produce a reparative cardiac patch. The use of aligned nanofiber scaffolds facilitated the alignment of CPC cells, and surface modification with gelatin or fibronectin improved metabolism. The conditioned media from these scaffolds reduced fibrosis gene expression, suggesting their potential therapeutic utility through paracrine activity [221].

In a cell‐based strategy, Rufaihah et al. proposed a synthetic glycosaminoglycan‐mimetic peptide nanofiber system that supports neovascularisation and cardiomyocyte differentiation, even in the absence of growth factors or stem cells. The injection of Rufaihah et al. nanofiber gels into infarcted rat hearts improved heart function, thereby providing a cell‐free approach for the repair of the myocardium [222]. Building on the development of nanofiber technologies, Liu et al. later developed a polymeric nanofiber bundle with a 3D architecture specifically for cardiac cell cultures. The bundles exhibited topological grooves that supported cardiomyocyte alignment and anisotropic contraction, as verified by SEM and immunofluorescence microscopy [223]. Suhaeri et al. developed a hybrid cell support composed of electrospun PLCL and fibroblast‐derived extracellular matrix (PLCL/FDM), which improved cardiomyoblast differentiation and cardiomyocyte development, using multi‐layer coculture systems that facilitated easy cell harvest and enhanced cell‐cell interactions, and performed better than regular coculture systems [224].

Wu et al. reported a new technique, 3D nanofiber‐assisted embedded bioprinting (3D‐NFEP), which utilises a 3D‐oriented nanofiber scaffold fabricated by divergent electrospinning, acting as a physical guide for cardiomyocytes’ alignment and confining the extrusion bioprinting of low‐viscosity hydrogels within limited spatial dimensions. Human‐induced pluripotent stem cells (iPSCs)‐derived cardiomyocytes (hiPSC‐CMs) were bioprinted onto a nanofiber support mesh using extrusion bioprinting. The cells attached to the array of aligned fibres after degradation of the hydrogel, which facilitated a high degree of alignment with minimal chemical modification of the hydrogel, a marked advantage over existing technologies. To produce multilayered cardiac tissue, the extrusion needle is adjusted to varying depths within the 3D nanofiber network. Figure 9Ia,b illustrates the existence of distinct differences in contour definition between top and bottom focal planes, which confirms the successful multi‐layer structure of the cells. Moreover, from Figure 9 Ic, it is evident that cells obtained from a monolithic GelMAdroplet were dispersed inside a 3D environment with a minimum thickness of 300 µm, which is owed to the supporting structure offered by the nanofiber scaffold. The ability to design thick, anisotropic cardiac tissue also indicates that 3D‐NFEP has the potential to be a versatile tool in cardiac tissue engineering [225].

**FIGURE 9:**
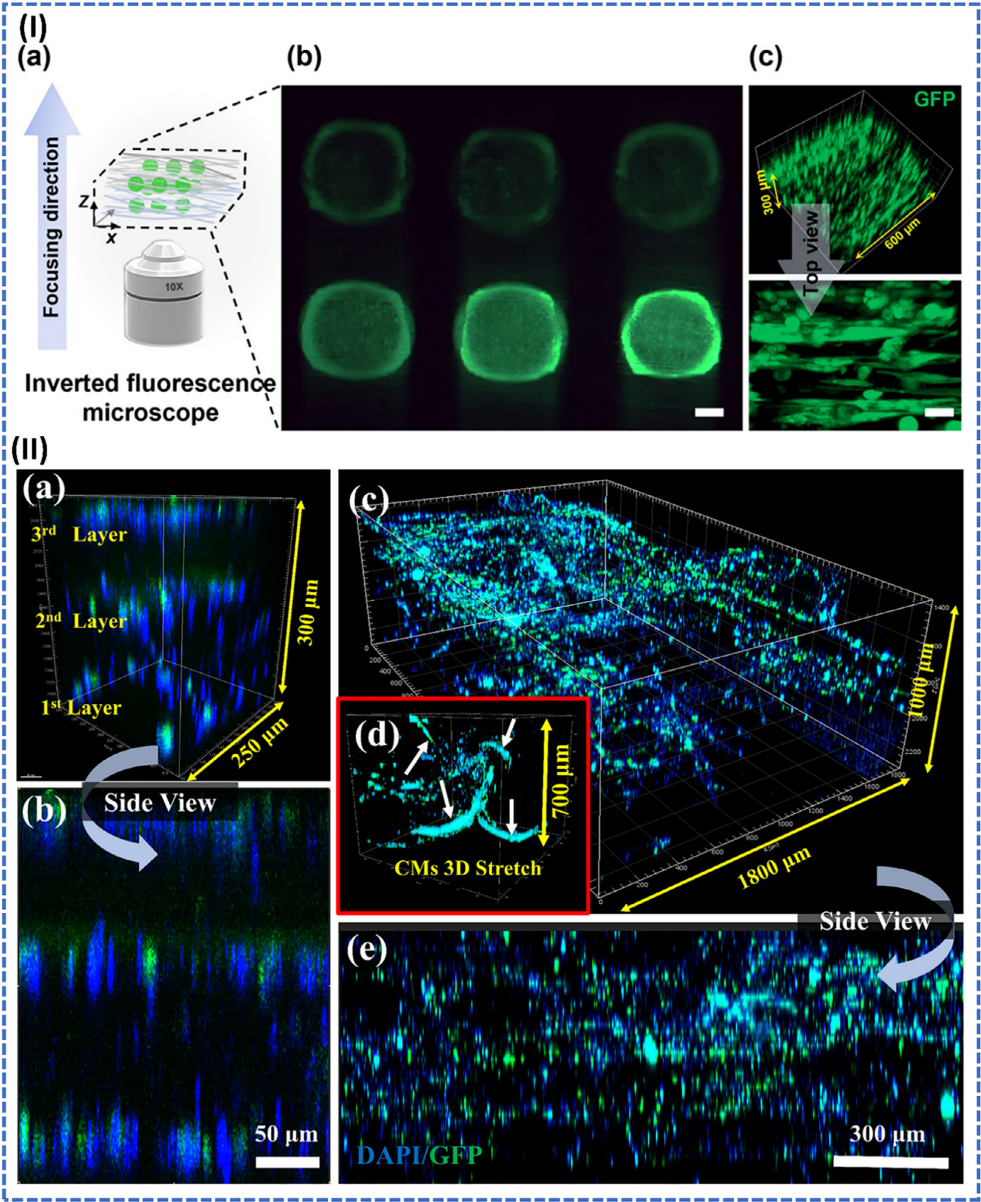
Application of Textiles and Nanofibers in Tissue Engineering Applications. (I) Regulation of multilayer cardiac tissue printing within 3D nanofiber scaffolds. (a) The diagram illustrates real‐time monitoring of sequential layer depositions during bioprinting. (b) Fluorescent images showing the distribution of autofluorescent cardiac tissue across different layers (scale bar = 200 µm). (c) Confocal image depicting the 3D organisation of cardiac tissue formed by individual droplets within aligned nanofibers (scale bar = 20 µm). Reprinted with copyright permission of [225], 2024 American Chemical Society. (II) Visualization of cardiomyocyte distribution within 3D nanofiber scaffolds: (a, b) multi‐layered scaffolds formed in trenches with a width‐to‐height ratio of 0.5, and (c–e) fused 3D nanofiber scaffolds created in trenches with a ratio of 1.5. Nuclei are stained blue (DAPI) and cardiomyocytes green (GFP). Reprinted with copyright permission of [226], 2024 American Chemical Society.

B. Qiu et al. developed a trench‐assisted electrospinning (ES) technique combined with direct ink writing (DIW) to produce a 3D‐aligned nanofiber scaffold with tunable porosities (60%–80%) and alignment (30%–60%). This has a trench width‐to‐height ratio of 1.5, which promotes alignment greater than 60%, ensuring uniform dispersion of hiPSC‐CMs in a ∼1000 µm‐thick layer. Additionally, it promotes cell infiltration and anisotropic growth compared to 2D ES. The results showed that scaffolds with a 0.5 ratio produced distinct layers at the interfaces (Figure 9IIa,b). In contrast, scaffolds with a 1.5 ratio yielded well‐distributed cells in 3D space (Figure 9IIc‐e). The attachment of cardiomyocytes to the scaffolds is evident in Figure 9IId, which shows cells extending along the suspended nanofiber cues, indicating improved cell attachment and morphology [226].

Taken together, these findings emphasise the importance of aligning nanofibers, surface‐modified nanofibers with biological molecules, and coupling them with microfluidic/ECM components to direct the alignment, development, and recovery of heart cells. The use of nanofibers, in addition to providing a platform for mimicking diseases in physiologically relevant conditions, offers a valuable platform for drug screening and regenerative therapy. Future research should focus on maximising the biological properties of scaffolds, scaling up, and developing cell‐free nanofiber technologies for minimally invasive cardiovascular repair.

## 7 Performance Tradeoffs of Smart Textiles for Wearable Cardiovascular Monitoring

Smart fabrics have emerged as a promising platform for wearable cardiovascular monitoring, with the seamless integration of sensors into everyday garments for continuous health tracking. Unlike rigid devices, these fabrics are designed to combine comfort, flexibility, and an unobtrusive design with unmatched sensing capabilities [227, 228]. Achieving this convergence poses significant challenges across material selection, signal fidelity, power management, and data security. Designers need to consider trade‐offs among various competing characteristics, including durability vs. comfort, accuracy vs. wearability, and cost vs. scalability. Furthermore, factors such as sweat, motion, and laundering further complicate long‐term reliability. Understanding these challenges and the inherent trade‐offs is crucial to developing practical, clinically relevant smart textile solutions for long‐term cardiovascular monitoring.

### 7.1 Material and Fabrication Challenges

Smart textiles designed for cardiovascular monitoring require the integration of functional materials such as conductive fabrics, flexible electrodes, micro‐encapsulated microelectronics, and even microfluidic or sensing layers with traditional fabrics. This comes with a range of challenges. First, it should be biocompatible for long‐term wear. The fibers, coating, adhesives, and finish should not cause irritation, dermatitis, or allergic reactions, especially when exposed to perspiration, heat, and friction. It is not straightforward to make it hypoallergenic while maintaining electrical conductivity because electrically conductive coating materials such as silver and nickel tend to leach or oxidize over time [228, 229].

Second, the system must be mechanically robust. The fabric will be subjected to cyclic loading (stretching, bending, and torsion), rubbing, and washing, while the conductive traces will be microcracked and delaminated, exhibiting changes in resistance. Methods for overcoming these include developing conductive core‐shell yarns, using serpentine‐mesh electrode layouts, and/or elastomeric encapsulation. Washing durability requirements, such as multiple machine‐wash cycles, various detergents, and different temperatures, are very challenging to meet while maintaining flexibility and sensor performance [230, 231, 232]. Conventional textile fabrication techniques, such as knitting, weaving, and braiding, must be adapted to functional materials. However, the assembly requirements for the electronic functions impose limitations on reflow temperatures, solvent exposure, and lamination force. Integration of all these requirements is often achieved through a process known as hybrid manufacturing, which involves roll‐to‐roll printing of electrodes, embroidery of interconnects, and low‐temperature soldering or conductive glue for electronic components [227, 233].

The main trade‐off is between functionality and comfort: the more robust and thicker the encapsulation and conductive traces are, the less breathable and stiffer the material becomes. Miniaturisation may help reduce size, but could make the material harder to restore and more brittle. Finally, scaling up is a problem: prototypes developed in the lab and produced at an industrial scale may be less likely to succeed and may require closer monitoring.

### 7.2 Signal Quality and Accuracy

Cardiovascular monitoring using smart textiles typically employs bioelectrical (ECG) and optical (PPG) sensing modalities. The fidelity of these signals depends on the quality of the electrode‐skin interface, optical coupling, motion resilience, and environmental stability. A significant challenge arises from motion artefact: locomotion, postural changes, and fabric micromotion cause fluctuations in contact impedance for ECG and in optical path length or pressure for PPG, which appear as baseline wander, amplitude distortions, and spurious peaks. The problem is aggravated by loose‐fitting garments and during vigorous activity [234, 235].

It is rather tricky to maintain constant contact between the electrodes and the skin without adhesive gels. The drawbacks of dry textile electrodes include high impedance and uneven compression, which adversely affect the signal‐to‐noise ratio and electromagnetic susceptibility. Some solutions to these disadvantages include the use of elastic compression areas, texture electrodes to maximise contact area, and humidity management to enhance impedance stability. However, increased compression improves signal quality at the expense of user comfort [230, 236].

On the processing side, rejection of artefactual data based on motion‐sensor Inertial Measurement Unit (IMU) fusion, adaptive filtering, or machine‐learning algorithms may substantially enhance accuracy. However, these approaches are often computationally demanding and can fail if the training set is unrepresentative. Calibrating for multiple individuals, different body types, and differing fitted garments is challenging [237, 238].

In fact, there is a trade‐off between clinical‐grade accuracy, which requires a good fit, higher‐quality electrodes, and more processing, and usability, wearability, and potential for longer battery life. A pragmatic approach is tiered monitoring, where well‐being metrics in casual wear, and diagnostic‐grade signals in purpose‐fit garments.

### 7.3 Power and Energy Management

There are trade‐offs between continued sensing, processing, and wireless communication with finite power resources. Traditional coin cells or thin‐film batteries are cumbersome and rigid, with short lifespans that hinder continuous monitoring [239]. Although ultralow‐power sensor interfaces or duty‐cycled operation could prolong battery life, these strategies may reduce sampling rates or reaction times for acute cardiovascular events such as arrhythmias or orthostatic hypotension [240, 241].

While new methods of harvesting energy such as thermoelectric (temperature gradients due to human body warmth) [242], piezoelectric (movement/strain) [243], triboelectric (contact electrification) [244], and photovoltaic (available ambient lighting) [245], are promising, they can provide intermittent power in the range of a few milliwatts per unit with a relatively low power density. System integration also raises new challenges: thermal interfaces need to be comfortable; piezoelectric or triboelectric components may alter the fabric's texture; and PV cells are not optimised for indoor use. Also, harvested power might need additional processing, rectification, DC‐DC conversion, and power management [241, 246, 247].

The other major issue is the design of power conditioning and distribution. The traces should have low resistance and be flexible. The power connection should withstand washing and bending. The methods of energy storage, micro‐supercapacitors, and thin‐film Li batteries involve a trade‐off between capacity and safety. When used to provide immersion resistance, encapsulation could increase temperatures and affect user comfort [248, 249, 250].

Performing edge computation (artefacts, features, events) on the device saves even more energy by reducing the amount of data that needs to be transmitted, but it also increases the computational load. Streaming actual sensor data can stress the radio, particularly during connection intervals in Bluetooth Low Energy (BLE) and Wi‐Fi. Where energy harvesting, waveform fidelity, and power management intersect, there are always trade‐offs between power and performance. For instance, larger batteries increase runtime but reduce flexibility. At the same time, higher compression ratios improve power efficiency but could compromise waveform fidelity, and more energy harvesting reduces reliance on batteries but adds complexity to system implementation. A multi‐tier system could ensure an optimal user experience [192, 251, 252].

### 7.4 Data Transmission and Processing

Smart textiles need to safely and efficiently transmit health information from the body to decision‐makers. Wireless standards such as BLE, ANT+, Wi‑Fi, and sometimes cellular modules offer a range of throughput and latency profiles, each with distinct power, interference, and coexistence considerations. BLE is preferred for its very low power consumption and integration with smartphones; however, its limited bandwidth makes it less than ideal for high‐fidelity multichannel streaming. Wi‑Fi enables much richer data transfer but increases energy consumption, network configuration complexity, and susceptibility to congestion in dense environments [253, 254, 255].

Reliable data transmission requires link‐resilience techniques such as channel hopping, retransmissions, and error correction. Interference from other wearables, earbuds, and home IoT networks might increase channel collision rates [256]. Antenna placement on flexible materials makes impedance matching harder. The body could shadow the antenna or increase the moisture on its surface. Antenna shielding increases reliability but may make clothing less flexible. On‐garment processing chains handle signal conditioning, such as filtering and baseline removal; feature extraction, including heart rate, HRV analysis, and morphology; and/or edge AI rhythm classification and anomaly scoring [254]. Edge computing capabilities reduce reliance on continuous transmission and promote privacy, as raw signals remain on devices. Edge computing capabilities have increased compute requirements, heightened the need for firmware updates, and enabled stronger generalization performance across users. The models face memory limitations due to wireless updates, which introduce security and versioning issues [253, 257, 258].

The trade‐offs center on utility vs. the expense of energy and complexity. More frequently, information‐rich data flows facilitate clinical insights but consume power and are difficult to implement on radios; higher levels of local processing and sensing improve both range and privacy concerns but intensify power and thermal constraints. Representative solutions employ hybrid approaches that combine low‐rate continuous telemetering with burst‐mode, high‐resolution transmissions in response to clinically relevant events.

### 7.5 Washability and Maintenance

In smart textiles, to succeed beyond the laboratory setting, washability and ease of use must be non‐negotiable attributes. However, the laundry process can introduce heat, chemicals, and mechanical forces that may damage sensors and affect their electrical properties. Soap can weaken polymer encapsulants, cause elastomers to swell, and corrode metalized layers; agitation can subject the material to cyclic forces; and dryer heat and ironing can introduce sufficient heat to cause delamination [259, 260]. Design considerations: hermetic encapsulation of electronics, conformal coatings (parylene, polyurethane, silicone), water‐resistant interconnects (crimped/laser‐welded joints, conductive sew paths), and textile electrodes that can tolerate wetting and drying without substantial changes in impedance. Binder materials for conductive ink must be detergent‐proof, whereas sew‐through conductive yarn requires core‐shell structures to prevent oxidation. Yet, increased thickness and protective overlays also decrease breathability, drape, and potentially wearability [260, 261, 262].

User‐friendly maintenance functions, such as removable electronic modules for washing, simple cleaning instructions, and compatibility with typical machine‐washing settings, are beneficial. On the downside, a module‐for‐module interface is susceptible to connector failures. While standards‐based testing may assess robustness, such as ISO/IEC washing tests, it might not reflect typical variations found in home laundry or typical home cleaners. Drying methods affect longevity: Tumble drying is more stressful than line drying; heat can accelerate the ageing of adhesives and batteries. Exposure to sweat between washes requires antimicrobial, moisture‐wicking layers that do not compromise electrode or dermatological compatibility. Drift‐correction errors after washing might require self‐testing or auto‐calibration procedures during garment donning [260, 263, 264].

Ultimately, designers are left with a triangle of issues: wash count resilience, comfort, and price. While the greatest wash‐count resilience may be achieved with higher‐cost, stiffer materials, this compromises washability. The use of detachable modules enhances washability but adds complexity to use.

### 7.6 Cost and Scalability

The challenge of scaling smart textile prototypes into cost‐efficient products lies in integrating the precision of electronics manufacturing with the volume and cost efficiency of textile manufacturing. While the existing cost of materials, such as conductive yarns (silver‐plated or stainless‐steel composites), speciality elastomers, encapsulants, and microcontrollers, remains higher than that of conventional textile components, the process cost further complicates matters [265, 266]. Defect‐related yield loss (broken traces, adhesion, alignment, and antenna detuning) may significantly increase the cost per unit, especially during the first production lots. Laundering and stress testing need to be incorporated into the QA process, which will add to costs and cycle time. The quality requirements for a medical‐grade product assure a redundant design, strict tolerancing, and a possible certification track (IEC/ISO, FDA/CE, depending on the claim), all of which increase non‐recurring engineering expenses.

From a supply chain perspective, integrating multi‐materials involves synchronising the supply chains for textiles, electronics, adhesives, and packaging materials. Lead times for non‐commercial conductive yarn materials and encapsulants may inhibit production processes. Companies have the dilemma of whether to integrate or partner in the manufacture of textiles and electronics [267, 268, 269]. Scalability is also dependent on automation. Although clothing is usually made by hand for fitness and detailing, smart textiles require automated module placement and machine embroidery to reduce variation. However, to justify investment in automation, demand forecasting must cover the costs. Modular designs, which break up the clothing and electronics pod, can make returns or upgrades easier, though they may be more complex from a BOM cost perspective [270, 271, 272]. Price sensitivity varies across markets: consumer wellness can tolerate lower accuracy at lower price points, whereas clinical applications justify higher costs to ensure validated performance. A practical approach is a platform‐based design with a shared sensor/electronics architecture across multiple garments, leveraging economies of scale while tiering features to match segment‐specific price elasticity.

### 7.7 Privacy and Security

Cardiovascular monitoring data collected continuously is highly sensitive and encompasses several aspects, including ECG waveforms, heart rate variability, arrhythmic alerts, activity levels, and associated context (timestamps and geographical locations from complementary devices). Data protection mechanisms for this data must be strongly privacy‐by‐design and security‐focused because it involve wearables, mobile applications, and the cloud [11, 273, 274].

At the device level, secure boot, firmware signing, and hardware‐backed keys help resist tampering and unauthorized code execution. Edge computation on the garment mitigates risks by transmitting only derived results, rather than raw waveforms, but models deployed for decision‐making should be both explainable and auditable to prevent bias or misclassification. Secure wireless communication, using methods such as authenticated pairing with session keys and encryption (BLE LE Secure Connections, TLS for mobile/cloud), can be provided. However, this requires stronger cryptography, which inherently increases computation overhead and energy consumption due to textiles' low‐power properties [275, 276, 277].

Mobile applications become a critical trust boundary: data minimization, transparent consent, granular sharing controls, and encryption of local storage protect users against overcollection and leakage. Push notifications and over‐the‐air updates should be authenticated to prevent spoofed alerts or malicious firmware. In the cloud, access control, audit logging, anonymization or pseudonymization, and compliance with relevant regulations (e.g., HIPAA and equivalent frameworks) would be essential to protect personal data. Role‐based access ensures clinicians see only what they should; de‐identification enables aggregate research while limiting the risk of re‐identification. The attack surface also includes lost or resold garments, compromised phones, insecure home networks, and third‐party integrations. Security key rotation, revocation, incident response, and vulnerability disclosure programs must be part of the product lifecycle. On the user side, technical controls are complemented by education on safe practices such as password security, operating system updates, and secure pairing [278, 279, 280].

Trade‐offs are unavoidable: increased security raises the power and latency budget and could make UX more complex. Overly constraining policies can hamper medical workflows. By employing a layered strategy (default private policies, context‐ and battery‐aware adaptive encryption policies, and transparent privacy control panels), it is possible to offer a fair trade‐off between protection and usability. Trust is also a product feature: transparency in privacy policies, independent third‐party security audits, and responsiveness to updates are as essential as sensor accuracy.

## 8 Future Trends and Outlook

Wearable cardiovascular systems based on smart textiles are entering a period of revolutionary transformation, driven by rapid convergence across materials science, bioengineering, artificial intelligence, and systems biology. The integration of edge‐enabled artificial intelligence, hybrid energy autonomy, Internet of Things (IoT) connectivity, advanced 2D and bio‐integrated materials, multi‐modal physiological sensing, and clinically validated digital health frameworks will define the next generation of smart textile platforms. Together, these advances will shift smart textiles from proof‐of‐concept demonstrations into clinically reliable, autonomous, and patient‐centric cardiovascular health products. This section outlines the key emerging directions and the interdisciplinary challenges that must be addressed to realize this transformative vision.

### 8.1 Artificial Intelligence Integration and Edge Intelligence

AI integration is among the most impactful near‐term trajectories for e‐textile cardiovascular platforms. Conventional signal‐processing pipelines for ECG, photoplethysmography (PPG), and pulse‐wave sensing are being replaced by lightweight machine‐learning models that run directly on low‐power microcontrollers embedded in garments. This paradigm, called edge AI or on‐device intelligence, offers transformative advantages: continuous, real‐time inference without reliance on cloud connectivity; reduced data transmission overhead; improved privacy by avoiding the upload of raw physiological waveforms; and faster alerting for acute cardiovascular events, such as arrhythmia onset or sudden changes in blood pressure. Edge AI solutions for the smart textile system will leverage techniques such as quantized neural networks and temporal models for microcontrollers. This will enable dynamic adjustment of sensing parameters based on activity or anomalies. This will provide greater diagnostic capabilities and decrease the rate of false positives. Another potential advantage is the use of federated learning for continuously improving model performance without compromising user privacy. In a federated architecture, each user's garment trains a local model update using their own physiological data, transmitting only gradient updates (rather than raw signals) to a central aggregation server. This approach enables population‐level model improvement while preserving data locality and privacy, a critical consideration given the sensitivity of cardiovascular health data and the regulatory requirements imposed by frameworks such as HIPAA and GDPR. Federated learning also offers the prospect of personalized model adaptation: as a garment accumulates a user's longitudinal cardiovascular data, local fine‐tuning can calibrate signal processing thresholds and detection models to account for inter‐individual variability in ECG morphology, skin impedance, and arterial pulse waveform characteristics.

A key challenge in deploying AI on smart textiles is the diversity and non‐stationarity of the signals themselves. Motion artefacts, electrode‐skin impedance variation due to sweat accumulation, sensor misalignment during dynamic activities, and signal degradation can introduce distribution shifts that can compromise model generalization. Future systems will need to incorporate adaptive signal‐quality assessment modules that detect and compensate for such artefacts in real time, flagging low‐confidence windows for exclusion or prompting the user to adjust the device. Looking further ahead, explainable AI (XAI) methodologies will be essential for clinical adoption. Cardiologists and regulatory bodies will require not only accurate predictions but also transparent reasoning that allows clinicians to verify, trust, and override automated diagnoses. The development of clinically interpretable AI frameworks tailored to the specific waveform morphologies and temporal patterns of textile‐acquired cardiovascular signals remains an important open research challenge.

### 8.2 Energy Autonomy

Power autonomy represents one of the most pressing unresolved engineering challenges in wearable cardiovascular textiles. Contemporary devices rely predominantly on thin‐film lithium‐ion batteries or lithium‐polymer cells, which impose trade‐offs among energy density, form factor, flexibility, washability, and recharge frequency. The vision of truly autonomous, battery‐independent cardiovascular monitoring garments requires a shift toward hybrid energy architectures that continuously harvest ambient energy from multiple bodily and environmental sources, efficiently store it, and manage its distribution to sensing, processing, and communication subsystems. The aim is to provide a nearly autonomous mode without restricting comfort and fashion. Currently, the challenge is designing the solution using these components without compromising the existing flexibility or the washing facility [21, 281, 282].

The human body provides multiple sources of energy. Thermoelectric generators (TEGs) exploit the temperature gradient between the skin surface and the ambient environment, converting it into electrical power via the Seebeck effect. While insufficient as standalone power sources for high‐duty‐cycle operation, thermoelectric textiles can serve as continuous background chargers for micro‐supercapacitors or thin‐film batteries during periods of low sensing demand. Future advances in organic thermoelectric materials could include composites with enhanced Seebeck coefficients, offering the prospect of washable, flexible, and high‐area‐coverage thermoelectric fabrics seamlessly integrated into cardiovascular monitoring garments.

Mechanical energy harvesting via piezoelectric and triboelectric transducers offers complementary capabilities, converting the kinetic energy of bodily motion, respiration, and arterial pulsation directly into electrical signals that simultaneously serve both sensing and power‐generation functions. PVDF‐based piezoelectric nanofiber textiles and triboelectric nanogenerators (TENGs) incorporating other materials and engineered architectures are driving the future of this field. The dual‐function nature of these transducers (simultaneously sensing and generating power from the same mechanical input) makes them particularly attractive for smart textile integration, enabling self‐powered pulse‐wave and respiratory monitoring without dedicated power rails.

Photovoltaic (PV) integration via flexible organic solar cell textiles could add a further dimension to hybrid harvesting. However, indoor light levels limit PV output to a few microwatts per square centimeter under typical office lighting.

Energy storage in the smart textile system is equally critical: wearable micro‐supercapacitors or batteries are essential for continuous monitoring.

The architectural challenge of integrating multiple harvesting modules, storage elements, and power management electronics into a single washable, flexible garment without compromising comfort, drape, or aesthetics remains substantial. Ultimately, the target architecture for next‐generation cardiovascular textiles will combine multiple harvesting pathways with intelligent power management to achieve near‐continuous operation over long wear durations without charging, representing a step change from current battery‐dependent paradigms.

### 8.3 Next‐Generation Materials

The materials landscape underpinning e‐textiles for cardiovascular monitoring is itself undergoing a significant transformation. Among the most promising material classes are MXenes, carbon nanotubes (CNTs), graphene (incl. reduced graphene oxide), PVDF‐TrFE, Galinstan, EGaIn, conductive hydrogels, magnetoelastic fibers, triboelectric nanogenerator materials, silk, cotton, cellulose, PDMS, Ecoflex, TPU, and spandex. Combining these materials into composites can yield new, promising materials with enhanced performance or multimodal sensing capabilities.

Innovative materials with high electrical conductivity and favorable mechanical properties (robustness, flexibility, elasticity) are paving the way for next‐generation e‐textiles with the potential for cardiovascular monitoring.

Future research could focus on improving the long‐term stability and biocompatibility of conductive materials in humid environments by encapsulating them in polymers (e.g., via coaxial electrospinning) or by surface functionalization.

Implantable biocompatible fiber systems represent a further frontier, extending the reach of textile electronics from the skin surface into the body itself. The development of biocompatible, biodegradable fiber electronics could enable implantable cardiovascular sensors that dissolve harmlessly after a defined monitoring window, eliminating the need for retrieval surgery. Advances in self‐powered vascular grafts that integrate triboelectric and/or piezoelectric sensing with wireless power transfer via near‐field communication (NFC) or inductive coupling could enable long‐term post‐operative hemodynamic monitoring following bypass surgery or endovascular repair, providing early warning of graft failure, anastomotic stenosis, or thrombosis.

### 8.4 Multi‐Modal Sensing

The cardiovascular system exhibits its pathophysiological states through a wide array of physical and biochemical signals. Current smart textile systems predominantly focus on one or two sensing modalities (usually ECG and pulse). The future smart textile platforms could be distinguished by their ability to simultaneously capture multiple signals and fuse them into comprehensive, high‐confidence cardiovascular health assessments.

Emerging multimodal textile architectures could integrate ECG electrodes, optical fibers, impedance plethysmography electrodes, piezoelectric pressure sensors, and biochemical sensors for sweat‐based biomarker analysis into unified garment platforms. Simultaneous measurement of multiple signals could be used to provide continuous blood pressure estimation via pulse transit time and to detect subtle changes in cardiac output and stroke volume that are invisible to any single modality. Impedance plethysmography, when integrated with ECG electrodes, could enable non‐invasive estimation of systemic vascular resistance and cardiac output, which is useful for heart failure management.

The addition of biochemical sensing layers opens the door to correlating cardiac electrical and mechanical signals with metabolic context. Sweat‐based electrochemical sensors integrated into textile substrates can monitor lactate, glucose, electrolytes (Na^+^, K^+^, Ca^2^^+^), and cortisol in real time, providing insight into cardiovascular risk factors. The development of multiplexed electrochemical arrays printed directly onto stretchable textile substrates, combined with microfluidic sweat‐collection channels woven into garment architectures, enables practical sweat biosensing during natural perspiration without the need for iontophoresis or sweat stimulation. Future integration of these biochemical sensing layers with simultaneous ECG and hemodynamic monitoring will enable unprecedented multidimensional cardiovascular phenotyping in daily life, with the potential to identify at‐risk individuals well before symptomatic cardiovascular events.

Multi‐modal sensor fusion requires sophisticated signal processing and data integration algorithms capable of handling signals with heterogeneous sampling rates, noise characteristics, and physiological information content. Machine learning models trained on multimodal input streams can achieve high accuracy in arrhythmia detection. They will likely become the standard computational framework for next‐generation clinical decision support in smart textile systems.

### 8.5 IoT‐Enabled Personalized Healthcare

The integration of smart cardiovascular textiles into broader IoT‐enabled health ecosystems will fundamentally redefine the relationship between patients, clinicians, and healthcare systems. Future smart textiles would seamlessly integrate with smartphones, home hubs, and cloud platforms, enabling continuous data sharing with healthcare professionals and facilitating remote patient monitoring initiatives, enabling health teams to monitor real‐time cardiac measures and respond appropriately. Eventually, smart textiles would help form digital twins, or digital replicas of human physiology, allowing for predictive models for health outcomes based on real‐time physiological data inputs, or personalized health recommendations [283, 284].

Achieving such future visions requires an interdisciplinary effort integrating materials science, electrical engineering, and data science. Engineers will need to co‐optimize hardware solutions and artificial intelligence algorithms to deliver reliable performance in real‐world settings, including motion, perspiration, and laundering. Also, hybrid power plans must strike a balance among autonomy, wearability, and lifetime. Software design will need to incorporate plans for interoperability, dynamic telemetry, and privacy‐preserving data analysis. Last, large‐scale validation research will play a pivotal role in determining the clinical dependability of such future visions. It will go on to encompass equity considerations in algorithm training datasets, thereby preserving equity. To close, the intersection of AI, EE, and IoT will transform smart materials into intelligent, self‐reliant, and networked systems in cardiovascular health. Smart materials will not only focus on health‐related physiological signal tracking, sensing, or detection. Rather, they will provide actionable forecasts, facilitate timely responses, and seamlessly integrate into more complex, tailored health plans. The future will see such enabling materials transition from emerging technologies to valuable health companions, accompanying an era of extensive, unobtrusive, and highly dedicated cardiovascular health monitoring.

### 8.6 Design Principles for Next‐Generation Cardiovascular E‐Textiles

Despite the intensive development of cardiovascular e‐textiles, a wide variety of approaches employ diverse materials, textile structures, sensing techniques, and system configurations. To move forward, it is necessary to establish universal design rules to create reliable, scalable, and clinically relevant systems.

First, cardiovascular monitoring systems must be designed according to the selected physiological parameter rather than material properties. For ECG monitoring, stable electrode‐skin contact and low contact impedance are needed. In contrast, for pulse wave, blood pressure, and hemodynamic measurements, high mechanical sensitivity and precise signal acquisition are required. Thus, the needs in clinical practice must be determined for the properties of the material, sensor, and garment.

Second, the electrode‐skin interface must become a primary design feature. In numerous papers, it has been shown that the quality of the signal depends much more on the stability of the interface, the conformability of the skin, and the contact pressure than on the conductivity of the electrode material.

Thirdly, the choice of textile architecture type should take into account the device's functions. Usually, knit fabrics offer greater elasticity and body conformity for continuous cardiovascular monitoring, whereas woven fabrics provide better electrical and geometric stability. Embroidered textile structures and combinations allow the incorporation of sensors at specific locations and provide design flexibility. In the future, it is necessary to consider textile architecture as part of the system that influences sensing results.

Another principle concerns the compromise between mechanical and electrical stability. Cardiovascular e‐textiles must be able to operate under constant stretching, bending and dynamic deformations. The stiffness of the system reduces wearability and user compliance; excessive flexibility, on the other hand, can reduce signal stability. Thus, optimal systems must combine wearability and sensing effectiveness.

Moreover, multimodal sensing technologies are expected to play an important role in the design paradigm for next‐generation cardiovascular monitoring systems. The use of ECG, photoplethysmography, pressure sensors, impedance plethysmography, and hemodynamic monitoring in a single textile sensor suit will provide greater insight into cardiovascular function.

The future of e‐textile technology must embrace a system‐oriented design paradigm which encompasses sensing, wireless communications, energy management, artificial intelligence, and data analytics. Clinical success will require not only material innovations but also the optimization of all these factors in parallel.

## 9 Conclusion

Smart textiles are emerging as a promising platform for continuous cardiovascular monitoring by seamlessly integrating sensing capabilities into comfortable, wearable garments. Recent advances have enabled textile‐based systems to monitor a wide range of physiological signals, including electrocardiography (ECG), pulse waveforms, blood pressure, and hemodynamic parameters, while maintaining flexibility, breathability, and skin conformity. These features support long‐term, unobtrusive monitoring in everyday settings and enhance user compliance compared with conventional wearable devices.

Despite significant progress, several challenges continue to limit widespread adoption, including motion‐induced artifacts, long‐term signal stability, durability in daily use, power management requirements, data security, and the lack of standardized evaluation protocols. Addressing these issues is essential for reliable real‐world implementation and clinical acceptance.

Looking ahead, the integration of artificial intelligence, edge computing, wireless connectivity, and self‐sustaining operation strategies is expected to improve signal accuracy, enable real‐time health analytics, and support personalized cardiovascular care. With continued interdisciplinary development, smart textiles have the potential to evolve into intelligent and clinically reliable systems for continuous, predictive, and patient‐centered cardiovascular monitoring.

## Author Contributions

**Bangul Khan**: Conceptualization, Writing – Original draft preparation, Writing – Reviewing and Editing, Talha Khalid: Writing – Original draft preparation. **Mahmoud Tavakoli**: Reviewing and Editing, Visualization and Validation. **Norbert Radacsi**: Reviewing and Editing, Visualization and Supervision. **Bee Luan KHOO**: Conceptualization, Writing – Original draft preparation, Writing – Reviewing and Editing, Visualization and Supervision.

## Funding

The authors have nothing to report.

## Declaration of Generative AI

During the preparation of this manuscript, the authors used the AI‐powered Grammarly software, licensed through City University of Hong Kong, to assist with grammar and language editing. All suggestions were reviewed by the authors, who take full responsibility for the manuscript's content.

## Conflicts of Interest

The authors declare no conflicts of interest.

## Data Availability

Data sharing not applicable to this article as no datasets were generated or analysed during the current study.
